# Lamin B1 regulates somatic mutations and progression of B-cell malignancies

**DOI:** 10.1038/leu.2017.255

**Published:** 2017-09-01

**Authors:** T Klymenko, J Bloehdorn, J Bahlo, S Robrecht, G Akylzhanova, K Cox, S Estenfelder, J Wang, J Edelmann, J C Strefford, T K Wojdacz, K Fischer, M Hallek, S Stilgenbauer, M Cragg, J Gribben, A Braun

**Affiliations:** 1 Centre for Haemato-Oncology, Barts Cancer Institute, Queen Mary University, London, UK; 2 Department of Internal Medicine III, University of Ulm, Ulm, Germany; 3 Department I of Internal Medicine, Center for Integrated Oncology Cologne, University Hospital of Cologne, Cologne, Germany; 4 Academic Unit of Cancer Sciences, Faculty of Medicine, Cancer Research UK Centre and Experimental Cancer Medicine Centre, University of Southampton, Southampton, UK; 5 Aarhus Institute of Advanced Studies, Aarhus University, Aarhus, Denmark

## Abstract

Somatic hypermutation (SHM) is a pivotal process in adaptive immunity that occurs in the germinal centre and allows B cells to change their primary DNA sequence and diversify their antigen receptors. Here, we report that genome binding of Lamin B1, a component of the nuclear envelope involved in epigenetic chromatin regulation, is reduced during B-cell activation and formation of lymphoid germinal centres. Chromatin immunoprecipitation-Seq analysis showed that kappa and heavy variable immunoglobulin domains were released from the Lamin B1 suppressive environment when SHM was induced in B cells. RNA interference-mediated reduction of Lamin B1 resulted in spontaneous SHM as well as kappa-light chain aberrant surface expression. Finally, Lamin B1 expression level correlated with progression-free and overall survival in chronic lymphocytic leukaemia, and was strongly involved in the transformation of follicular lymphoma. In summary, here we report that Lamin B1 is a negative epigenetic regulator of SHM in normal B-cells and a ‘mutational gatekeeper’, suppressing the aberrant mutations that drive lymphoid malignancy.

## Introduction

Class-switch recombination and somatic hypermutation (SHM) are biological mechanisms through which B cells adapt and respond to pathogens. These mechanisms involve a regulated process of targeted mutation within the variable regions of immunoglobulin genes, thus diversifying the antibody repertoire and allowing affinity maturation and isotype class switching. There is increasing evidence that epigenetic factors, such as DNA methylation and post-translational histone modifications, have major roles in regulating class-switch recombination and SHM.^1^ In addition to regulating expression of the central mutating enzyme activation-induced cytidine deaminase (AID), these epigenetic factors (for example, Spt6, H2B^Ser14p^) also target the SHM/class-switch recombination machinery, in a manner independent of V(D)J or S region transcription, by inducing an open chromatin state and recruiting critical adaptor proteins.^2, 3, 4^ Thus, B-cell SHM can be regulated by a higher order of chromatin organisation.

From that perspective, it is intriguing that the subnuclear position of chromatin domains (that is, proximity to the nuclear envelope) has been suggested to impact both transcription and V(D)J recombination.^5^ Furthermore, peripheral interphase relocalisation of immunoglobulin variable regions during both B-cell development^5^ and antibody production in plasma cells,^6^ suggests an ‘*in situ*’ epigenetic mechanism by which sub-telomeric (IgHV) or peri-centromeric (IgKV and IgLV) immunoglobulin variable loci can transition from repressive to permissive chromatin states to facilitate rearrangement.

The nuclear periphery, containing the IgH and IgK gene clusters,^5, 7^ is a unique compartment comprised of inner nuclear membrane proteins and nuclear lamina.^8^ Previous genome-wide and cytological studies revealed the regulatory role for some of these nuclear proteins in higher level genome organisation and gene regulation.^9^ In particular, lamina-associated domains (LADs) were identified at the nuclear periphery using the DNA adenine methyltransferase identification (DamID) technique.^10^ Initial reports described LADs as large (0.1–10 Mb), transcriptionally silent, gene-poor domains associated with Lamin B1, comprising of up to a quarter of nuclear chromatin.^10^ More recent studies have also revealed an important role of LADs in the regulation of gene expression and recombination.^11, 12^ Moreover, developmentally regulated genes were found to be specifically enriched in these domains,^13, 14^ leading to the theory that LADs are regulated as facultative heterochromatin compartments during development. In agreement with this, large-scale chromatin relaxation and aberrant transcription were specifically linked to Lamin B1 depletion in senescent fibroblasts and progeria cells.^15, 16^ Furthermore, age-associated loss of Lamin B1 has been reported to lead to systemic inflammation in Drosophila due to the derepression of a large number of immune responsive genes.^17^ These data strongly suggest the restrictive role of LADs in epigenetic gene regulation. This restrictive influence can be transient and tightly regulated depending on the cellular differentiation state.

Topologically, centromeric and telomeric chromosome regions were the primary candidates for lamina-mediated epigenetic gene regulation as both were shown to colocalise with intra-nuclear lamina structures resulting in their preferred peripheral distribution.^18, 19^

Given the apparent topological coincidence between LADs and Ig variable clusters, we hypothesised that nuclear lamina might have a paramount role in the dynamics of Ig-encoding variable genome clusters. In particular, here we tested whether Lamin B1, a principal LAD-associated component of the nuclear envelope, had any restrictive role on SHM and the expression of Ig genes. Due to the strong involvement of IgV mutations in the pathogenesis of B-cell malignancies, we also tested whether nuclear lamina is involved in the pathogenesis of germinal centre (GC) lymphomas and chronic lymphocytic leukaemia (CLL). Finally, we have elucidated associations of Lamin B1 expression with other prognostic factors in CLL and its impact on the disease course in a front-line clinical treatment trial (CLL8 study).

## Materials and methods

### Cell lines

Pfeiffer, Raji and SU-DHL4 cells were obtained from American Type Culture Collection (ATCC). EHEB and Karpas422 cell lines were obtained from European Collection of Authenticated Cell Cultures (ECACC, Public Health England). BL2 cell line was obtained from the German Collection of Microorganisms and Cell culture (ACC 625. Deutsche Sammlung von Mikroorganismen und Zellkulturen, DSMZ). BL2 AID-/- cells were kindly provided by Claude-Agnes Reynaud (INSERM U1151, Paris) and were described previously.^20^ Cells were maintained in antibiotic-free RPMI 1640 medium with fetal calf serum (10% Sigma-Aldrich, Gillingham, UK) and glutamine (2 mM; Gibco, Invitrogen, Renfrew, UK) at 37 °C, 5% CO_2_, and were routinely screened for mycoplasma contamination. Cytogenetically, BL2 cells were characterised as human flat-moded near-diploid karyotype with 3% polyploidy; 44–47<2n>XY, der(1)t(1;7)(q32;q11.2), der(6)t(1;6)(q21;q25), t(8;22)(q24;q11.2), del(11)(q24.2); carrying t(8;22) effecting juxtaposition of MYC with IGL@. Immunophenotypically, cells were CD3−, CD10+, CD13–, CD19+, CD20+, CD34−, CD37+, CD38+, cyCD79a+, CD80+, CD138−, HLA-DR+, sm/cyIgG−, sm/cyIgM+, sm/cy kappa−, sm/cy lambda+.

### Induction and quantification of SHM in the Ig V gene

*In vitro* SHM was induced as described^20^ with minor modifications. Cells were incubated at 2 × 10^6^ cells/ml in RPMI medium, containing 2.5 μg/ml of biotinylated anti−human IgM (clone UCHB1 Caltag Laboratories, Buckingham, UK), 10 ug/ml of anti-CD19 (clone RFB9, in-house, Southampton, UK) and 10 ug/ml of anti-CD21 (clone HB135, in-house, Southampton, UK) for 20 min at 4 °C. Cells were washed and then resuspended in RPMI medium containing streptavidin-conjugated magnetic beads (5−7 beads/cell) (Dynabeads M280, Thermo-Fisher, Renfrew, UK) and incubated with agitation at 4 °C for 20 min. Complete RPMI medium containing 10% FBS was added to the activated cells to a final density of 1 × 10^6^, followed by incubation at 37 °C for 24, 48 or 72 h. To analyse Ig gene hypermutation, the *V4-39-JH5* gene was amplified from genomic DNA with Pfu DNA polymerase (Thermo Scientific). The primers used were Vh4-forward 5′-TTCTTCCTCCTGCTGGTGGCG-3′, Jh5 reverse 5′-CTCCCCGGCTTTCTTTCCTG-3′. The conditions for PCR amplification were 94^0^ for 30 s, 60^0^ for 30 s, 72^0^ for 75 s, 25 cycles. The PCR products were then gel-purified with a QIAquick gel extraction kit (Qiagen, Manchester, UK) and cloned with the Zero Blunt TOPO PCR cloning kit (Thermo Fisher Scientific, Renfrew, UK). Plasmid DNA extracted from individual bacterial colonies was sequenced in an automated sequencer. Mutations per base pair were calculated after aligning the V4-39-JH5 sequence from treated cells to the reference sequence (Supplementary Figure 2) using DNASTAR's SeqMan NGen software. At least 10 000 base pairs were assessed per experimental condition.

### Chromatin immunoprecipitation (ChIP)-sequencing analysis of lamin B1 binding

ChIP was performed as described.^16^ BL2 cells were crosslinked with 1% paraformaldehyde for 5 min at room temperature. Paraformaldehyde was then quenched with glycine, and cells were harvested and sonicated using Bioruptor Plus (Diagenode, Seraing, Belgium) 5–8 cycles 30 s active/30 s inactive pulses to produce soluble ~300 bp chromatin fragments. Lamin B1 and control IgG ChIP-Seq was performed on two independent biological replicates with corresponding inputs per each condition (control and SHM-induced), and then antibody-bound chromatin was immobilised with anti-rabbit IgG-conjugated Dynabeads (112.04D, Invitrogen). DNA libraries were prepared using Illumina Nextera DNA Library Preparation Kit (FC-121-1030), and then massive parallel sequencing was performed using Illumina HiSeq2500 sequencer, yielding ~70 mln to 90 mln raw reads per sample.

### Massively parallel sequencing and bioinformatical data analysis

Raw reads were mapped to the human genome (hg19) using the Bowtie 2 alignment software.^21^ Alignment BAM files were sorted by coordinates, and PCR duplicates were removed using Picard's MarkDuplicates program. To avoid any normalisation bias, each pair of aligned input and ChIP BAM files were further processed to have the same read depth, using Picard's DownsampleSam program on the larger of the two files. Lamin B1 enriched regions were defined using the enriched domain detector algorithm as described.^22^ The aligned peaks were then visualised using the Interactive Genome Browser (IGV, Broad Institute, Cambridge, Massachusetts).^23^ Complete ChIP-Seq data sets are available at Gene Expression Omnibus (GEO) with accession number GSE89869.

### LMNB1 RNA interference (RNAi) experiments

For LMNB1 RNAi transfections, 50 nM short interfering RNA (siRNA) was electroporated into 10^6^ of logarithmically growing BL2 cells using Lonza Nucleofector 2b device (Basel, Switzerland), nucleofection solution T and C009 programme. After electroporation, cells were resuspended and immediately seeded into a pre-warmed fresh growth medium at 0.25 × 10^6^ cells/ml concentration. Total and incorporated Lamin B1 levels were assessed by WB or ImageStream cytometry 48 h after electroporation. siRNAs used for this study: siGENOME siRNAs were received from Dharmacon (GE Healthcare, Roserberg, Sweden) and target sequences were the following: siRNA1—5′-GAAGGAAUCUGAUCUUAAU-3′, siRNA2—5′-CAACUGACCUCAUCUGGAA-3′, siRNA3—5′-GAAAGAGUCUAGAGCAUGU-3′, 5′-GCAUGAAACGCGCUUGGUA-3′. ON-TARGETplus Non-targeting Control siRNA Pool (D-001810-10-05, Dharmacon) was used at 50 nM as an off-target siRNA control.

### Microscopy and image analysis

For immunofluorescence microscopy, harvested cells were cytospun onto poly-l-lysine–coated microscope slides (suspension cells) or mouse spleens were cryosectioned using Leica CM3050 cryostat. Slides were fixed in –20 °C methanol and then rinsed briefly in ice-cold acetone. Slides were washed in tris buffer saline (TBS)/0.05% Tween and then incubated with appropriately diluted primary antibodies for 60–120 min at room temperature. The primary antibodies were diluted in TBS/0.1% bovine serum albumin. Subsequently, slides were washed × 3 in TBS-Tween and stained using the anti-rabbit Alexa Fluor 488, anti-mouse Alexa Fluor 594 or anti-rat Alexa Fluor 594 (all Abcam, Cambridge, UK). After three washes in TBS/0.05% Tween-20, DNA was counterstained with propidium iodide or 4',6-Diamidino-2-Phenylindole, Dihydrochloride before mounting in ProLong Gold Antifade Reagent (Invitrogen). Images were collected using a Nikon Ci-L upright fluorescence microscope and NIS elements software. *In situ* fluorescence intensity was measured within the linear fluorescence range, using MetaMorph software and integrated morphometry analysis module.

Primary antibodies used for immunofluorescence: rabbit anti-Lamin B1 (Abcam, ab16048), mouse anti-CD27 (BioLegend, London, UK, 356401), rat anti-mouse IgD (Southern Biotech, Cambridge, UK, 1120-01), rat anti-CD45R (B220) (Abcam, ab64100).

### High-throughput image stream cytometry

For image stream cytometry, cells were fixed in −20 °C methanol, washed × 3 in PBS and then non-specific binding was blocked using a 5 ml of PBS/0.05% Tween/2% fetal calf serum. Cells were then stained rabbit anti-Lamin B1 antibody (Abcam, ab16048) in PBS/Tween/2% fetal calf serum for 60 min. The concentration of primary antibody was calibrated to keep the acquired fluorescence within the linear dynamic range. After three washes, cells were then stained with anti-rabbit Alexa Fluor 488, washed again, and then DNA was counterstained with 4',6-Diamidino-2-Phenylindole, Dihydrochloride. High-throughput images were acquired using ImageStream X Mk II imaging cytometer, and Lamin B1 parameters were analysed using Excel software.

### Flow cytometry of plasma membrane surface immunoglobulins

To label cell surface immunoglobulins, cells were washed in PBS and resuspended in PBS/5% fetal calf serum containing fluorochrome-labelled anti-Ig antibodies. After 1 h incubation, cells were washed in PBS, fixed in 2% paraformaldehyde for 20 min, washed again and assessed by FACScalibur analyser (BD Biosciences, Oxford, UK). Isotype and fluorochrome-matched non-targeting antibodies, added at equal concentrations, were used to set the background fluorescence. The following BioLegend (BioLegend UK, London, UK) antibodies were used in this study: PE anti-human Ig light chain λ (316607), PE Mouse IgG2a, κ isotype Ctrl (FC) (400213), PE anti-human Ig light chain κ antibody (316507), PE Mouse IgG1, κ isotype Ctrl (FC) antibody (400113), FITC anti-human IgD antibody (348205), FITC Mouse IgG2a, κ isotype Ctrl (FC) antibody (400209). FITC Fab2 anti-human IgG (F0185), RPE Fab2 anti-human IgM (R5111), and corresponding isotype controls (control reagent, Rabbit F(ab')2/FITC, X0929, and control reagent, Rabbit F(ab')2/RPE, X0930) were obtained from Dako (Agilent Technologies, Cheadle, UK).

### Cell proliferation assay

Cell proliferation was assessed by FACS using Click-iT Plus EdU Flow Cytometry or by fluorescence microscopy using Click-iT EdU Alexa Fluor 488 Imaging Kits (Invitrogen/Thermo-Fisher, Paisley, UK). In all, 10 μM EdU was added to growing cells for 2 (LMNB1 siRNA experiments) or 16 (assessment of steady-state proliferation) hours and then cells were processed essentially according to the manufacturer’s protocols.

### Western blotting

Whole-cell lysates were prepared in NuPage LDS sample buffer (Thermo-Fisher), containing 0.1 M DTT. Samples were then separated by using 4–12% or 10% pre-cast NuPAGE Novex gels (Thermo-Fisher), and proteins were transferred onto polyvinylidene difluoride membrane using a wet transfer system (BioRad, Hemel Hempstead, UK). Membranes were blocked with 5% non-fat dried milk in TBS/0.05% Tween, incubated with the appropriately diluted primary antibodies, washed, incubated with horseradish peroxidase–conjugated anti-rabbit, anti-mouse IgG (GE Healthcare/Amersham, Little Chalfont, UK) or anti-rat (DAKO/Agilent, Stockport, UK), and visualised by ECL (GE Healthcare/Amersham, Little Chalfont, UK) and ChemiDoc imaging system (BioRad, Hemel Hempstead, UK). Primary antibodies used for WB: rabbit anti-Lamin B1 (Abcam, ab16048), rabbit anti-histone H3 (Abcam, ab1791), mouse anti-Bcl6 (Active Motif, 61194), Rat anti-AID (Active Motif, 39886).

### Animal immunisation

C57BL/6 mice were immunised by IP injection with 10 ng LPS plus 0.5 mg ovalbumin (SIGMA) in 200 ul PBS. In all, 7 or 14 days later mice were killed, spleens removed and processed for IHC/IF or the homogenised and the GC B cells were isolated by MACS using a Mouse Germinal Center B Cell (PNA) MicroBead Kit (Miltenyi Biotec, Bisley, UK). Animals were maintained in local facilities and experiments approved by local ethical committees under Home Office license PPL30/2964.

### Taqman quantitative reverse transcription PCR (qRT-PCR) gene expression analysis

For cell line, genomic DNA and total RNA were purified using AllPrep DNA/RNA mini kit (Qiagen) as per the manufacturer’s instructions. Total RNA from primary B-cells isolated from mouse spleens was purified using RNeasy mini kit. To remove DNA contaminations, all RNA samples were treated on-column with the QIAGEN RNase-Free DNase; purified RNA was quantified with Qubit RNA BR (Broad-Range) Assay Kit (Thermofisher) and reverse transcribed using High-Capacity RNA-to-cDNA Kit (Applied Biosystems/Thermo Fisher Scientific, Renfrew, UK) according to the manufacturer’s instructions. Triplicate amplification reactions containing 15–50 ng or input RNA each were carried out. TaqMan probes used in this study are listed in Supplementary Table 1. mRNA encoding ribosomal protein S18 and β-actin were used as the standard internal controls. Reaction mixes (20 μl) contained TaqMan Gene Expression Master Mix 1 ×, TagMan Gene Expression Assay 1 × and template cDNA. qPCR reactions were performed and analysed on an Applied Biosystems ABI QuantStudio7 using ddCt comparison method. Graphing and statistical analyses were performed using GraphPad Prism 7. All three groups were compared with each other by one-way analysis of variance with Tukey’s post-test.

### Patients and samples

Expression profiling was conducted on peripheral blood samples from 337 previously untreated CLL patients. Samples were collected at enrolment on the CLL8-trial, a prospective, international, multicenter trial comparing first-line treatment with FC or FCR in a 1:1 randomised fashion (www.clinicaltrials.gov NCT00281918) as previously described.^24^ Ficoll density gradient centrifugation for isolation of mononuclear cells followed by an immunomagnetic tumor cell enrichment via CD19 (Midi MACS, Miltenyi Biotec, Bergisch Gladbach, Germany) was performed on all samples. Data on genomic aberrations (del(11)(q22.3), +12, del(13)(q14), del(17)(p13), t(11;14)) the IGHV, TP53, SF3B1 and NOTCH1 mutational status was assessed as previously described.^24, 25^ Written informed consent and local ethics committee approval was obtained in accordance with the Declaration of Helsinki for all patients.

Gene expression profiling on BL2 cell line was conducted for two independently cultured passages with four experimental approaches each, with (1) control with non-targeting siRNA (2) control with non-targeting siRNA and after induction of SHM (3) LMNB1 RNAi transfected (4) LMNB1 RNAi transfected and after induction of SHM. For siRNA-treated samples, SMARTpool LMNB1 siRNA mixture of three siRNAs in equimolar concentration was used.

### RNA isolation and quality assessment

Total RNA for mRNA profiling was extracted from whole-cell lysates of treated BL2 cells and primary patient samples according to the Allprep DNA/RNA mini kit (Qiagen). Quality control was assessed using the Agilent 2100 Bioanalyser with the RNA 6000 Nano LabChip (Agilent Technologies). The Chip was prepared according to the manufacturer's protocol and analysed using the 2100 Expert software. To secure the best accuracy and reproducibility samples with an RNA integrity number <7.0 were excluded from further analysis. RNA for gene expression profiling on BL2 cell line functional studies had a RNA integrity number of 10.

### Gene expression profiling on Exon ST 1.0 Arrays

Patient samples and samples from functional studies on BL2 cells were analysed for mRNA expression using the Affymetrix GeneChip Human Exon 1.0 ST Array (Affymetrix, Santa Clara, CA, USA). The experiment was conducted according to the manufacturer’s protocol. In brief, 250 ng RNA per sample were amplified, transcribed to cDNA, fragmented and subsequently labelled with Biotin. Array hybridisation was performed at 45 °C for 16–18 h in the Affymetrix GeneChip Hybridisation Oven 640, arrays were subsequently washed in the Fluidics Station 450 and scanned on the GeneChip scanner 3000 7G. Complete GEP data sets are available at Gene Expression Omnibus (GEO) with accession number GSE98529.

### Normalisation and analysis of expression data

Statistical procedures were performed with the R software version 2.15.1 and BRB-ArrayTools Version: 4.2.1 Raw Affymetrix Human Exon array (HuEx-1_0-st-v2) data files have been preprocessed by the robust multichip average algorithm using either the implementation in the R-package *oligo*^26^ or *aroma.affymterix* (University of California, Berkeley 2008). Besides robust multichip average normalisation the default background correction and quantile normalisation was applied. *Aroma.affymterix* was applied to generate gene expression values summarised on the exon/probe set level and on the transcript level using the ‘core’ probe set definition according to Affymetrix. Hierarchial clustering on selected genes was conducted using the ‘Genesis platform’^27^ using pearson correlation and average linkage as agglomeration rule and distance metric, respectively. Gene set enrichment analysis^28^ was performed on ‘C7: immunologic signatures’ gene sets compiled at the Molecular Signatures Database, Broad Institute.

### Statistical methods

Standard statistical means were used to evaluate associations between clinical and molecular patient characteristics (*χ*^2^/Fisher’s exact; Mann–Whitney/Kruskal–Wallis). Time-to-event parameters included progression-free survival (PFS) and overall survival (OS), and were estimated by the Kaplan–Meier method. Survival times were compared via two-sided non-stratified log-rank tests. Hazard ratios were calculated using Cox proportional-hazard regression analyses. All tests were two-sided, and a *P*-value <0.05 was defined as statistically significant. No adjustments for multiple tests were performed. Statistical analyses were performed with SPSS v23 (SPSS, Chicago, IL, USA).

## Results

**Figure 1:**
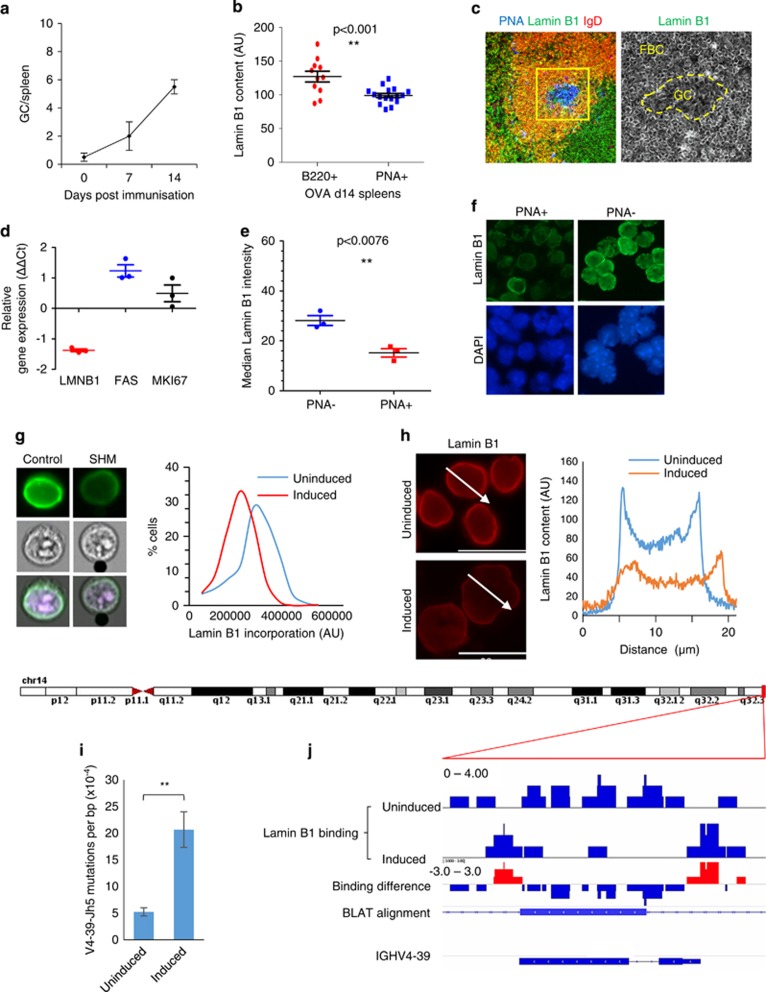
Nuclear Lamin B1 is reduced in follicular germinal centre B cells and is associated with somatic hypermutation. (**a**) C57BL/6 mice were immunised with LPS plus ovalbumin (OVA), and 7 or 14 days later spleens were removed and processed for immunohistochemistry. Figure represents the average number of GCs per spleen (*n*=3)±s.e.m. (**b**, **c**) Spleen GCs were stained with anti-Lamin B1 antibodies (AlexaFluor 488, green channel), Peanut Agglutinin (PNA) (Pacific Blue, blue channel), and anti-B220 or anti-IgD antibodies (AlexaFluor 594, red channel). Fluorescence intensity of the Lamin B1 channel (C, bottom image) was measured using MetaMorph image analysis software within (PNA+ areas) or outside (B220+ or IgD+ PNA- areas) the GCs. At least 10 GCs from three spleens were analysed, and their Lamin B1 fluorescence was compared with a similar number of randomly selected extra-GC areas of the same size. (**d**–**f**) C57BL/6 mice were immunised with LPS plus ovalbumin (OVA), and after 14 days, spleens were removed, and PNA+ cells were isolated using PNA positive separation MicroBead Kit. (**d**) Taqman qRT-PCR analysis showing relative *LMNB1*, *TNFR6* and *Mki67* mRNA level changes in PNA+ vs PNA- splenocytes. (**e**) After microbead separation, nuclear Lamin B1 incorporation was measured by *in situ* cytometry in PNA+ vs PNA- cells (*n*=3). (**f**) Representative Lamin B1 immunofluorescence (AlexaFluor 488, green) in PNA+ and PNA− cells in OVA immunised animals. Nuclei were counterstained with DAPI (blue). (**g**) SHM was induced in BL2 cells according to the protocol (see Materials and Methods) and nuclear Lamin B1 was assessed 48 h post induction by IF and ImageStream image cytometry. Total nuclear Lamin B1 levels were reduced by an average of 20%. (**h**) Line scan analysis by MetaMorph reveals an average 53% reduction in nuclear envelope bound Lamin B1 in SHM-induced cells, as compared with control cell population. (**g**, **h**) are the representative images of five independent experiments. (**i**) DNA from control or activated BL2 cells was isolated, cloned and then analysed for mutations within the *IGHV4-39* gene as described in Materials and Methods. The reference IGHV4-39 sequence and nucleotide substitution pattern after SHM induction and LMNB1 RNAi are displayed in Supplementary Figure 5. At least 10 000 base pairs (bp) were analysed per condition, and the mutational load was expressed as mutations per bp. *n*=4, ±s.e.m. *P*<0.01. (**j**) ChIP-Seq analysis of the dynamics of Lamin B1 binding to the *IGHV4-39* gene following SHM induction. As compared with control cells, Lamin B1 binding to this gene was significantly reduced 48 h after cell activation. BLAT alignment represents the *IGHV4-39* sequence where SHM was assessed by Sanger sequencing and clonal analysis. DAPI, 4',6-Diamidino-2-Phenylindole, Dihydrochloride.

To evaluate Lamin B1 dynamics in secondary lymphoid tissues, we performed IHC staining for Lamin B1 in spleens of OVA-challenged mice on day 14 post-immunisation, when the amount of GCs per spleen was maximal (Figure 1a), accompanied by an average of sixfold increase in GL7+ splenocytes (Supplementary Figures 1A and B). We observed a consistent reduction of nuclear Lamin B1 within peanut-agglutinin (PNA) visualised GC cells but not mantle zone B cells (Figures 1b and c) or follicular T cells (not shown).

Complementary to spatial analysis of Lamin B1 in spleens of OVA-immunised animals, we then MACS separated PNA+ splenocytes and performed qRT-PCR analysis of LMNB1 gene expression in PNA+ vs PNA- fractions. Figure 1d demonstrate that mRNA levels of LMNB1 were invariably decreased in PNA+ fraction. In contrast, expression of both *mKI67* and *TNFR6* were elevated in PNA+ cells, consistent with their biological function in activated murine B cells. Next, we tested whether reduction of *LMNB1* gene expression (Figure 1d) translates into a reduced nuclear incorporation of Lamin B1 protein in PNA+ splenocytes. Figures 1e and f, and Supplementary Figure 1C clearly demonstrate that, as compared with PNA-depleted fraction, nuclear Lamin B1 fluorescence is significantly decreased in PNA+ cells.

To test whether the decrease in Lamin B1 was a general cross-species phenomenon related to GC formation, we next assessed a panel of tissue microarrays containing 42 human biopsies with previously diagnosed reactive lymphoid hyperplasia. Microscopic studies, accompanied by image analysis revealed a consistent decrease in nuclear Lamin B1 within the intrafollicular areas (as assessed by Ki67 staining) of reactive lymph nodes, compared with extrafollicular IgD+ mantle zone areas (Supplementary Figures 1D and E). These results demonstrate a general decrease in Lamin B1 during GC formation in humans and mice.

We next hypothesised that the observed decrease in GC Lamin B1 might be associated with a specific change in GC B cells. After antigen engagement, activation and several rounds of cell division, B cells undergo SHM; a process by which the antibody-encoding DNA sequences are mutated at a highly elevated frequency, generating a diverse series of clones in the GC. We, therefore, tested whether induction of SHM was associated with a decrease in nuclear Lamin B1. For this purpose, we used BL2 cells, a human B-cell line used previously as a model for assessing SHM.^20^ Parallel assessment of Lamin B1 nuclear incorporation by ImageStream and *in situ* cytometry revealed a robust reduction of Lamin B1 at the nuclear periphery in cells undergoing SHM (Figures 1g and h).

Keeping in mind the previously reported role of Lamin B1 in the LAD-mediated epigenetic regulation of chromatin, we hypothesised that Lamin B1 might be a mutational ‘gatekeeper’, maintaining the IgH V gene clusters within the restrictive facultative heterochromatic regions. Upon antigen stimulation, these genome segments can be released from the suppressive environment of the nuclear lamina, followed by access for transcription and, potentially, mutations. In support of this hypothesis, analysis of previous chromatin immunoprecipitation (ChIP) studies performed in primary fibroblasts^15, 16^ revealed that LADs precisely co-localise with all three (kappa, lambda, heavy) Ig variable gene clusters (Supplementary Figure 2). This observation suggests that IgV gene clusters can be prime candidates for epigenetic regulation via LADs.

To address our hypothesis more directly, we next performed ChIP-sequencing analysis of Lamin B1 genome binding in the human BL2 cells undergoing SHM. Our analysis revealed that in control cells the topology of Lamin B1 binding (defined as LADs according to our previously used algorithms^29^) precisely coincide with the IgV clusters. Induction of SHM resulted in a rearrangement of Lamin B1 binding to the genome with a general drop of its binding to chromosomes 1 to 14 and increased binding to smaller chromosomes 15 to 22 (Supplementary Figure 3A). On that background, Lamin B1 ‘canyons’—areas of decreased genome binding—invariably coincided with IgV gene clusters, even if surrounded by ‘mesas’—areas of increased Lamin B1 binding after SHM induction (Supplementary Figure 3B).

Most importantly, results displayed in Figures 1i and j indicate that, after stimulation, mutations in a 565 bp reporter fragment of the V4-39-J_h_5 gene increases ~4.5-fold (Figure 1i and Supplementary Figure 4). Furthermore, 85% of nucleotide substitutions are consistent with AICDA-dependent cytidine deamination within the WRCY motif, a functional hallmark of SHM (Supplementary Figure 4C). This increased mutational load (Figure 1i) was accompanied by a gross threefold, yet precise reduction of Lamin B1 binding to this fragment after SHM induction (Figure 1j). These data suggest a direct involvement of Lamin B1 in epigenetic regulation of SHM in IgV domains.

We next suggested that the decreased nuclear Lamin B1 could be associated with altered cell cycle redistribution, which is a function of the proliferative capacity of cells. According to this hypothesis, cells that proliferate fast would have lower levels of nuclear Lamin B1, which in turn would be consistent with a higher proliferation rate of GC B cells *in vivo*, or activated BL2 or primary B cells *in vitro*. To address this hypothesis directly, we compared Lamin B1 levels within different cell cycle phases by FACS and immunofluorescence. Furthermore, we tested the correlation between the proportion of EdU-positive cells and their Lamin B1 level in six different cell lines under various growth conditions (Supplementary Figure 5). These experiments revealed no association between steady state cell proliferation and nuclear Lamin B1 (Supplementary Figure 5). In fact, more rapidly proliferating cells had a tendency to increase their Lamin B1 incorporation (Supplementary Figure 5A) due to the marginally higher amount of Lamin B1 in G2, as compared with G1 cells (Supplementary Figures 5B and C). Likewise, *in situ* cytometry showed no difference in Lamin B1 incorporation between EdU-positive and EdU-negative cells (Supplementary Figures 6D and E). These results suggest that a decreased Lamin B1 in GC B cells is likely to be associated with a more specific role, which is not a passive reflection of cellular proliferation *per se*.

**Figure 2:**
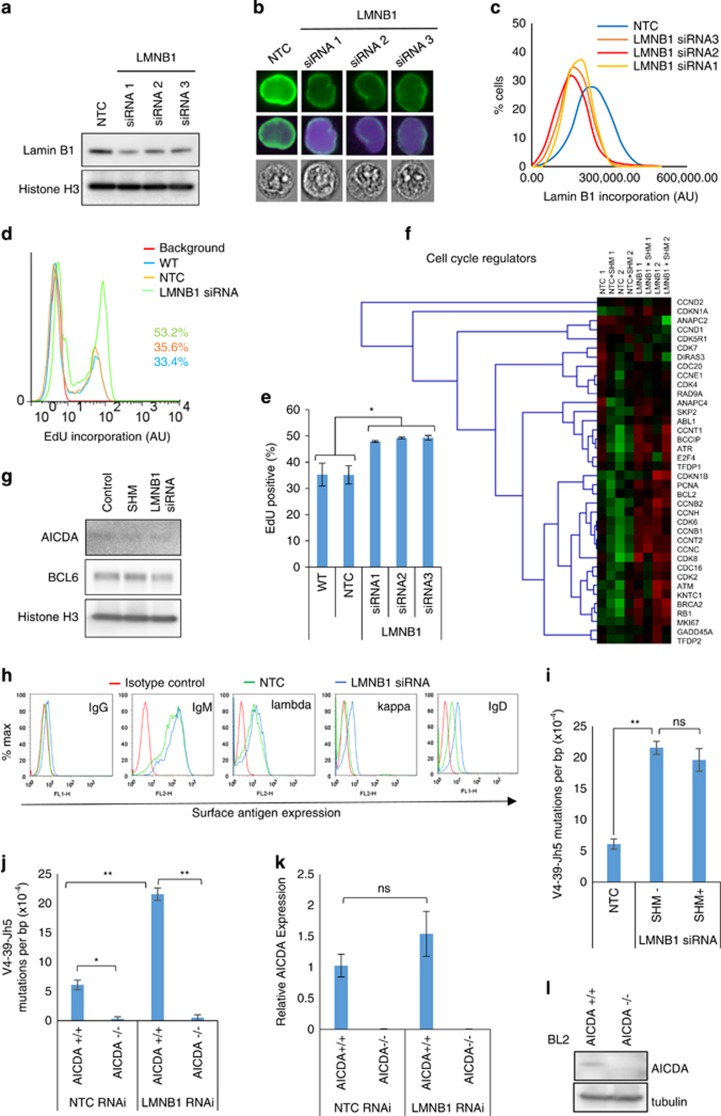
RNAi-mediated reduction of nuclear Lamin B1 results in acute proliferation boost, *de-novo* surface antigen expression and AICDA-dependent somatic mutations. (**a**) Three different LMNB1 mRNA-targeting siRNAs were nucleofected into BL2 cells and then total Lamin B1 protein level was assessed by western blotting 48 h post electroporation. (**b**, **c**) High throughput Lamin B1 nuclear incorporation was assessed by ImageStream cytometry 48 h after siRNA treatment. At least 8000 cells were analysed per sample. Nuclei were counterstained with DAPI. (**b**) Representative cells showing Lamin B1 fluorescence within the median of sample intensity distributions. (**c**) Histograms showing absolute value distributions for non-targeting siRNA control (NTC) or LMNB1 siRNA-treated cells. (**d**, **e**) EdU was added to WT, NTC or siRNA-treated cells 72 h post siRNA treatment for 2 h and then the proportion of EdU+ cells was assessed by FACS. (**d**) Representative EdU FACS profile showing 33% proliferation boost in siRNA2, as compared with NTC-treated cells. (**e**) The average percentage of EdU+ cells from three independent experiments±s.e.m. *P*<0.02. (**f**) Expression profiles of cell cycle regulatory genes in SHM-induced and/or LMNB1 siRNA-treated BL2 cells. (NTC: control with non-targeting siRNA; NTC SHM: control with non-targeting siRNA and after induction of SHM; LMNB1: LMNB1 RNAi transfected; LMNB1 SHM: LMNB1 RNAi transfected and after induction of SHM. Experiments were conducted in two independent BL2 cell passages indicated with ‘-1’ and ‘-2’ behind the experiment label. For siRNA-treated samples, a SMARTpool LMNB1 siRNA mixture of three siRNAs (**a**–**f**), in equimolar concentration was used. (**g**) Western blot analysis of AID and BCL6 proteins in antibody- or LMNB1 siRNA-treated samples at 72-hour time point. Histone H3 was used as a loading control. (**h**) FACS analysis of Ig surface antigen expression. Surface IgG, IgM, IgD, as well as kappa and lambda-light chain expression was assessed 72 h after cells were treated with NTC or SMARTpool LMNB1 siRNA. For FACS, a fluorophore and isotype-matched non-targeting antibody was used to set background fluorescence. A representative of three independent experiments. (**i**–**l**) Mutations targeted to V4-39-Jh5 fragment are AICDA dependent. (**i**) Cells were treated with SMART pool LMNB1 siRNA and then mutations were assessed by Sanger sequencing 72 h post transfection. For combined antibody/siRNA-treated samples, SHM was induced 48 h after LMNB1 siRNA transfection, and then DNA was isolated 72 h after initial treatment. (**j**) Parental and AID-deficient BL2-clones were treated with LMNB1 RNAi and mutations induced in V4-39-Jh5 gene were analysed. *P<0.05, **P<0.01. (**k**) qPCR and (**l**) western-blot analysis of AICDA expression in BL2 wt and AID-/- cells treated with non-target control (NTC) or LMNB1 siRNA. Data represents the average of three experiments±s.e.m. *P*<0.01.

To test the impact of Lamin B1 on cell dynamics and SHM directly, we then transfected BL2 cells with siRNA-targeting mRNA transcripts from three different exons of the *LMNB1* gene (See Materials and Methods). In general, 50–80% reduction of Lamin B1 protein level (Figure 2a) 48 h after siRNA electroporation translated to an average of 32% reduction in Lamin B1 nuclear incorporation (Figures 2b and c). Interestingly, this level of reduced Lamin B1 incorporation was very similar to that produced by SHM induction (compare Figures 1g and 2c), potentially suggesting a functional epigenetic compartment of nuclear Lamin B1 different from its structural function.

Having established a specific RNAi-mediated reduction of nuclear Lamin B1, we next tested whether Lamin B1^low^ cells would exhibit any phenotypic features characteristic of GC B cells. Our findings displayed on Figures 2d and e demonstrate that proliferation boost, as detected by on average 27% more EdU-positive cells, was a characteristic feature of siRNA-treated Lamin B1^low^ cells. This proliferation boost was accompanied by a general upregulation of positive cell cycle regulatory genes which, in turn, occurred alongside an upregulation of genes responsible for cell cycle checkpoint execution or cell cycle arrest, and were specific for LMNB1 reduction; independent of SHM induction (Figure 2f and Supplementary Figure 6). We believe the latter can be interpreted as a secondary cellular response to uncontrolled proliferation and DNA lesions.

AICDA is a master regulator of antibody diversification at DNA level via SHM and class-switch recombination.^30^ AID expression peaks in GCs where it is positively regulated by BCL6 via an indirect miR-155-mediated mechanism.^31^ We set out to examine that upregulation of AID might be a primary mechanism for SHM induction by antigen ligation or LMNB1 siRNA. Figure 2g shows that neither AICDA nor its GC regulator BCL6 is induced by antibodies or LMNB1 siRNA. We, therefore, suggest that Lamin B1-mediated chromatin accessibility could be the primary factor regulating SHM in BL2 cells.

It is suggested that individual B-cells in lymphoid tissues possess either kappa or lambda light chains. A mixture of kappa and lambda-positive cells is characteristic of reactive or otherwise benign GCs. Our previous immunophenotypic analysis of BL2 cells revealed strong lambda chain expression on the cell surface, which is likely to be due to BL2-specific t^8, 22^ translocation involving IGL@ locus. On the contrary, the non-rearranged kappa-light chain, expressed from chromosome 2, was largely absent from the cell surface. Consistent with our model, we found that IGK@ was heavily incorporated within the Lamin B1-binding sites. We next hypothesised that siRNA-mediated reduction of Lamin B1 incorporation would release the IGK@ locus from its suppressive environment that would result in *de novo* expression of kappa-light chain on the cell surface. In agreement with this hypothesis, decreasing Lamin B1 binding to the IGK@ locus (Supplementary Figure 3B) was accompanied by ~5-fold induction of kappa light chain expression on the cell surface (Figure 2h).

Finally, we tested whether siRNA-mediated reduction of Lamin B1 incorporation results in spontaneous SHM. By analogy with Figure 1i, clonal analysis of the IGHV4-39 locus revealed a ~4-fold induction of spontaneous SHM in cells treated with smartpooled LMNB1 siRNA (Figure 2i) with 72.4% of nucleotide substitutions falling within the AICDA-related mutational hotspots (Supplementary Figure 4D). Notably, combining LMNB1 siRNA treatment and induction of SHM by ligating surface antigens did not alter the SHM rate induced by siRNA-treatment alone. This suggests a shared cellular mechanism of SHM induction between nuclear lamina and external stimuli, resulting in a binary induction of SHM.

To further our functional evidence on the involvement of Lamin B1 in SHM, we next tested whether IGHV4-39 mutations occurring after LMNB1 RNAi treatment are AICDA dependent. To do this, we did similar LMNB1 RNAi treatments in AICDA-/- cells described previously.^20^ Figure 2j demonstrates that AICDA-/- background almost completely negates both background and LMNB1 RNAi-induced mutations. Interestingly, although we couldnot detect any AICDA signal by qRT-PCR and western blotting (Figures 2k and l), significant amount of AICDA expression could be observed in wt BL2 cells, which is in agreement with previously published reports.^20, 32^ Furthermore, it’s expression did not significantly change after LMNB1 RNAi (Figure 2k) that, in combination with Figure 2g, suggests that other chromatin factors are at least as important in SHM as AICDA expression *per se*. Out data suggest that lamina-mediated conformational changes could be one of these factors.

We were unable to establish a stable LMNB1-negative cell line using CRISPR/CAS9 or short hairpin RNA approach. Neither are we aware of the existence of any such human cell models, outside of cellular senescence.

**Figure 3:**
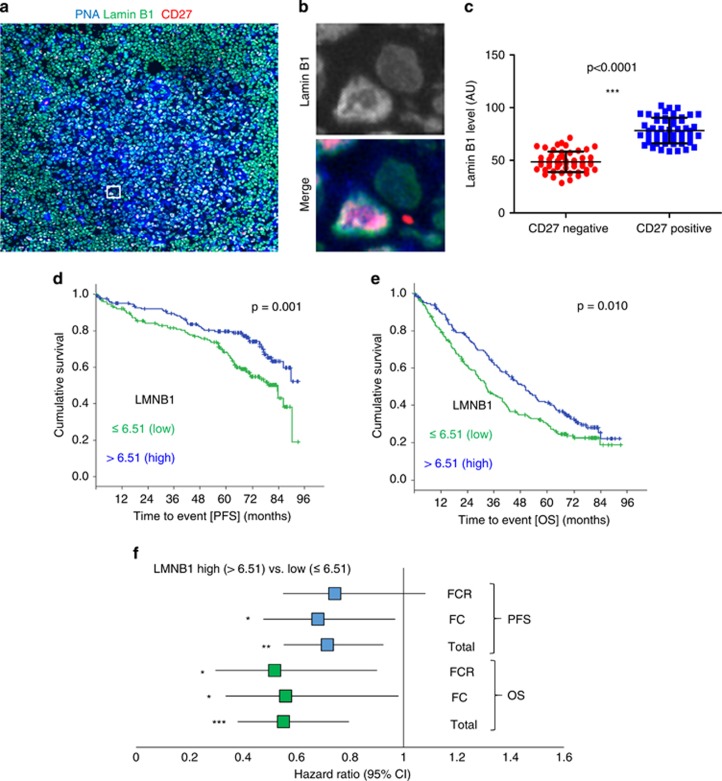
Nuclear Lamin B1 levels are restored in memory B cells and low LMNB1 expression level is an adverse prognostic factor in CLL. (**a**) Fresh frozen normal human lymph nodes were fixed in −20 °C methanol and then stained with PNA (blue), Lamin B1 (green) and anti-CD27 antibodies (red). (**b**) A zoomed in the area of (A, square) representing differential Lamin B1 expression in CD27+ vs CD27- cell. (**c**) High-throughput comparison of Lamin B1 levels in CD27+ and CD27- GC cells. Lymph nodes from three patients were assessed and in total 50 randomly selected CD27+, and 50 randomly selected CD27- GC cells were analysed using MetaMorph software. (**d**, **e**) showing Kaplan–Meier estimates of progression-free (PFS) (**d**) and overall (OS) (**e**) of CLL patients enrolled on the CLL8-trial as a factor of LMNB1 expression, dichotomised by the median (>6.51, *N*=168 and ⩽6.51, *N*=169. (**f**) Univariate Cox regression analysis comparing low (⩽6.51, *N*=169) and high (>6.51, *N*=168) LMNB1 expressing CLL patients as a factor of the treatment regime (FC or FCR) applied. *P<0.05, **P<0.01, ***P<0.001.

Formation of plasma or memory cells is the outcome for B cells following SHM and clonal selection.^33^ Given the acute functional impact of Lamin B1 in our *in vitro* system, we tested whether the drop in Lamin B1 in GC B cells translates into their ultimate differentiated state. If the GC-associated drop in Lamin B1 is transient, this might then suggest a temporary chromatin access for a naive B cell to rearrange its Ig domains and, hence, fine-tune the antibody repertoire. To address this question, we first compared the expression of Lamin B1 in human CD27^+^ vs CD27^−^ B cells within the GCs of fresh frozen human lymph nodes. CD27 is a widely accepted marker of memory B cells that can be detected in human GCs^34^ before GC-dependent memory cells relocate to the marginal zone.^35, 36^ Figures 3a–c demonstrates that CD27^+^ cells have a substantially higher expression of Lamin B1 compared with CD27^−^ GC B cells. These data suggest that the drop in Lamin B1 is transient and is only temporally associated with SHM in normal B cells.

Although the cellular origin of CLL is still debated, several lines of evidence suggest that CLL cells are antigen-experienced,^37^ resembling memory B cells.^38^ Within this context, IGHV-mutated CLL cells, associated with favourable clinical prognosis and derived from the CD5+/CD27+ post-GC B-cell subset, could be similar to ‘classic memory B cells’ generated by a typical GC-based reaction, represented in our system as a CD27+ GC B cells (Figures 3a–c).

By analogy with the normal CD27+ memory B cells, we hypothesised that IGHV-mutated CLL (mCLL) would be associated with higher Lamin B1 expression levels as compared with unmutated CLL cases (uCLL). Furthermore, given an association of IGHV mutational status with clinical outcome, we anticipated that Lamin B1 would *per se* constitute a prognostic factor in CLL.

With that in mind, we analysed gene expression profiles of 337 previously untreated CLL patients, enrolled on the CLL8 trial evaluating FC versus FCR in a randomised fashion (NCT00281918) (refs 24, 39). As implicated from our functional studies, higher *LMNB1* expression, as dichotomised at the median (LMNB1 low⩽6.51 vs LMNB1 high>6.51), was inversely correlated with high-risk genomic aberrations (Supplementary Table 2) and was associated with shorter median PFS (32.4 vs 49.9 months, *P*=0.010) and, notably, OS (83.6 months vs not reached, *P*=0.001) and (Figures 3d and e).

Conversely, we were unable to detect a similar clinical impact for LMNB2 and LMNA genes (Supplementary Figure 7), encoding two other components of the nuclear lamina, Lamin B2 and Lamin A/C, respectively. The latter highlights a highly specific impact of Lamin B1 in the molecular pathology of CLL.

Moreover, univariate Cox regression analysis comparing low (⩽ 6.51) and high (> 6.51) *LMNB1* expression cohorts revealed an overall hazard ratio of 0.715 for PFS and 0.551 for OS (95% confidence interval) (Figure 3f), suggesting a strong protective impact of *LMNB1* expression in CLL. Interestingly, further stratification of patients according to their treatment regime revealed a very similar *LMNB1*-related hazard ratio regardless of the therapeutic modality applied (Figure 3f). The latter suggests that the molecular mechanisms responsible *LMNB1*-mediated protection of CLL patients are different from the mechanisms covered by chemotherapy.

Further, we were able to solidify functional model-derived clinico-biologic implications in the context of CLL. First, we correlated *LMNB1* expression from our data sets with the total amount of Lamin B1 protein, and, although not absolute, a positive correlation between gene expression and protein content could be observed (Supplementary Figure 8A). Next, when performing gene set enrichment analysis for CLL cases dichotomised for lower and upper quartiles of *LMNB1* expression, we found ‘*LMNB1* quartile low’ cases to extensively match signatures of anti-IgM-activated B cells (Supplementary Figure 8B) while there was only a 1.12-fold expression change for AID observable for ‘*LMNB1* quartile low’ expressing CLLs (data not shown). This suggests that CLL cells with *LMNB1* downregulation are transcriptionally locked in an activated state. Complementary to this, and resembling *LMNB1* siRNA phenotype in BL2 cells, we found an inverse relationship between expression of *LMNB1* and expression of positive cell cycle regulatory genes (Supplementary Figure 8B). On that background, low *LMNB1* expression was also strongly associated with high *BCL2* and low *CDKN1A* expression levels (Supplementary Figure 8B), further highlighting similarities between Lamin B1^low^ and GC B cells.

We next assessed whether differential expression of *LMNB1* in CLL can be attributed to differential CpG methylation within this gene. Given a strong association of *LMNB1* expression with IGHV mutational status, we next compared the methylation values (normalised Infinium HumanMethylation450 BeadChip beta values) for *LMNB1* promoter CpG sites between CLL patients with mutated and non-mutated IGHV. Two sample *t*-test with equal variances revealed no association between *LMNB1* methylation and IGHV mutational status, suggesting that mechanisms other than CpG methylation are responsible for regulating *LMNB1* expression in CLL (Supplementary Figure 9).

**Figure 4:**
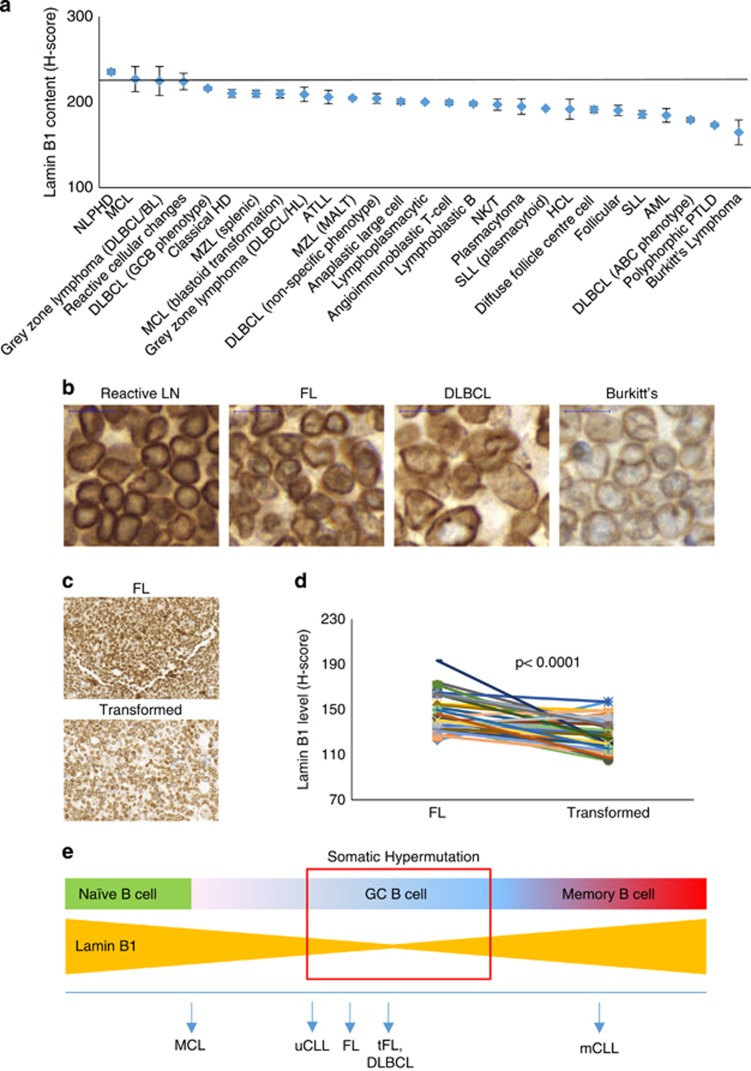
Nuclear Lamin B1 decreased in B-cell lymphomas. (**a**) Immunohistochemical tissue microarray analysis of nuclear Lamin B1 in tissue biopsies of B-cell malignancies showing decreasing LMNB1 staining with increasing aggressiveness. (**b**) Representative IHC images from the lymphoid tumour panel displayed in **a**. The horizontal line represents Lamin B1 immunoreactivity within the intrafollicular areas of benign reactive lymph nodes. At least three samples were analysed per tumour type, and bars represent average H-score values±s.e.m. (**c**) Lamin B1 immunoreactivity in sequential biopsies from a FL patient (upper image) that later underwent FL transformation (lower image). (**d**) Lamin B1 H-score values of 43 FL biopsies and their transformed counterparts. Each line connects two sequential biopsies from the same patient. (**e**) Lamin B1 dynamics within the context of B-cell natural development in secondary lymphoid organs. Upon antigen stimulation in secondary lymphoid organs, mature naive B cells undergo activation accompanied by a series of complex processes including SHM and clonal expansion. Our data suggest that specific reduction of nuclear Lamin B1 in GC B cells is instrumental for these processes. Furthermore, our observations suggest that this low level of Lamin B1 is locked in B cell malignancies arising from a corresponding B-cell development stage.

We next hypothesised that a permissive chromatin state, associated with decreased nuclear Lamin B1 in GC B cells, might be linked to secondary ‘off target’ mutation events. The latter can be followed by a formation of malignancies that originate in the GC such as follicular lymphoma (FL) or diffuse large B-cell lymphoma. In support of this hypothesis, we found a consistently decreased amount of Lamin B1 in the majority of primary lymphoid tumours, as compared with intrafollicular areas of normal human reactive lymph nodes (Figures 4a and b). Intriguingly, we found decreased Lamin B1 in other non-lymphoid malignancies including acute myeloid leukaemia demonstrating that LADs are also deregulated in other haematological malignancies (Figure 4a). Next, we assessed a chronological series of biopsies from 43 patients with FL, which subsequently underwent transformation. We found that FL transformation was strongly associated with a further decrease in Lamin B1 (Figures 4c and d), suggesting a possible involvement of LADs in the progression of this malignancy.

## Discussion

One of the principal questions in B-cell biology yet to be answered is how SHM machinery accesses immunoglobulin loci. For SHM to take place, Ig variable loci should be subjected to AID-mediated deamination as well as DNA cleavage and repair. Each of these events is likely to be regulated by specific changes in chromatin, resulting in its open and accessible conformation. In resting B cells, most of the IgH locus exist in a closed chromatin state, enriched in repressive histone marks, such as H3K9Me3 and H3K27Me3,^40^ as well as HP1-γ protein.^41^ Within that context, it is intriguing that both H3K9Me3 and HP1-γ are known to be associated with Lamin B1,^42^ topologically defining lamina-associated chromatin domains.^10, 16^

In this study, we show that perinuclear Lamin B1 is decreased in GCs of mouse and human lymphoid follicles. The reduction of nuclear Lamin B1 could also be observed after induction of SHM *in vitro*. This was accompanied by the reduced genome binding of Lamin B1, including the domains that encode variable immunoglobulin parts. Furthermore, the clonal analysis revealed that the rate of SHM was grossly increased when Lamin B1 genome incorporation was suppressed by RNA interference, providing for the first time a direct functional link between SHM and structural components of the nuclear envelope. Downstream of GC, we found that Lamin B1 levels were restored in CD27+ memory B cells indicating the temporary nature of Lamin B1 decrease in activated B cells. This suggests an ‘epigenetic window of opportunity’ for a GC B cell to start and complete SHM when the chromatin state is permissive.

Interestingly, Lamin B1 dynamics in GC/post-GC B cells translates into CLL, which is a post-GC malignancy. In particular, IGHV-mutated CLL cells, associated with a favourable clinical prognosis and derived from the CD5+/CD27+ post-GC B-cell subset^43^ displayed significantly higher *LMNB1* gene expression as compared with uCLL samples. Furthermore, low *LMNB1* expression was strongly associated with multiple cytogenetic abnormalities and was a strong-negative prognostic factor for both PFS and OS. The strong direct relationship between the *LMNB1* expression quartiles and survival (both OS and PFS) was independent of the treatment applied, suggesting a novel mechanism of molecular pathogenesis of CLL, which is beyond control by the currently available CLL treatment modalities. Targeting LaminB1-associated mechanisms in CLL may provide another step towards the post-chemotherapy era and complement existing therapies, potentially gaining even larger therapeutic margins for this disease. As low *LMNB1* expression was strongly associated with clinically adverse cytogenetic abnormalities, it is also tempting to speculate that, due to its proximal role in the nuclear structure and function, Lamin B1 may serve as a safeguard against chromosomal aberrations during the clonal evolution of CLL.

Deletions of 13q and 11q represent the most frequent and co-occurring aberrations in CLL^25^ and loss of the *DLEU2/miR-15a/16-1* cluster on 13q14 in mice is sufficient to initiate B-cell lymphoproliferative disorders with CLL-like phenotypes.^44^ Moreover, deletion 13q and trisomy 12, followed by deletion 11q, have been identified as early drivers in the evolutionary process of CLL.^45^ Therefore, we hypothesise that the observed significant drops in Lamin B1 binding upon induction of SHM especially on chromosomes 13 and 12, or with slightly lesser extend on chromosome 11, may constitute the central selective vulnerability for deleterious hits initiating the development of CLL.

In line with this suggestion, defects of Lamin B1 expression and processing have previously been linked to aberrant interphase chromosome positioning^46^ and chromatin instability^47^—a potential path to chromosomal aberrations. However, a direct functional link between *LMNB1* expression and chromosomal abnormalities is yet to be established.

Here, we also show that nuclear Lamin B1 is decreased in the majority of GC-derived lymphomas. Furthermore, sequential biopsies from individuals with transformed FL showed a noticeable decrease of perinuclear Lamin B1 content during FL transformation. The latter, unlike in CD27+ memory B or mCLL cells, suggests a GC-based clonal selection of Lamin B1^low^ B cells with their subsequent propagation into lymphoma cells, presumably due to increased mutational load associated with more permissive chromatin state. From a translational perspective, reduced levels of Lamin B1 in FL could be developed into a robust molecular biomarker predicting transformation of indolent lymphoma. At the moment, despite major advances in technology, no biomarker other than histologic grade has proven to be sufficiently robust to be widely accepted clinically.^48^

Overall, here we propose that Lamin B1 is an upstream epigenetic regulator of SHM in GC B cells. Functionally, activation of GC B cells is associated with a drop in Lamin B1 and release of unrearranged IgV chromatin domains from the restrictive influence of LADs, in turn, enabling normal SHM to occur. Finally, we suggest that this permissive chromatin state also increases the likelihood of ‘off target’ hits that ultimately contribute to the formation and progression of GC lymphomas and, at the post-GC level, CLL. Figure 4e represents a principal schema, outlining the Lamin B1 dynamics in normal and malignant B cells.

Having suggested a functional involvement of Lamin B1 in SHM regulation and tumour progression, there is a number of questions that are still outstanding and warrant further investigation. Firstly, it will be important to understand how Lamin B1 is regulated within the GCs. Our data suggest that differential mRNA expression could be one of these factors. However, given a rather rapid reduction of Lamin B1 incorporation during SHM induction in BL2 cells (in our system, as early as 90 min), one would suggest that post-translational regulation, could be equally involved in the reduction of nuclear Lamin B1. Within that context, it is possible that Lamin B1 phosphorylation by cdk1 and protein kinase C^49^ is one of the mechanisms for the rapid disassembly of nuclear Lamin B1 in GC B cells. Next, as we were unable to identify DNA methylation as a source for the differential *LMNB1* expression in CLL, establishing this mechanism would provide a novel insight into the pathophysiology of this disease, with clear translational potential.

Finally, taking into account clear involvement of Lamin B1 in the pathophysiology of GC and post-GC B-cell malignancies, manipulating nuclear Lamin B1 levels could provide a novel therapeutic approach to these tumours, beyond the treatment modalities based on chemotherapy or monoclonal antibodies. Within this context, we believe that Lamin B1 phosphorylation and farnesylation would be the primary therapeutic targets to control the dynamics of nuclear lamina in leukaemia and lymphoma.
