# Supplementary material for: Lamin B1 regulates somatic mutations and progression of B-cell malignancies
Source: Leukemia. 2017 Sep 1;32(2):364–75. doi: 10.1038/leu.2017.255 (PMC5808072; doi:10.1038/leu.2017.255)
Supplement: Supplementary Figures [file leu2017255x1.pdf]

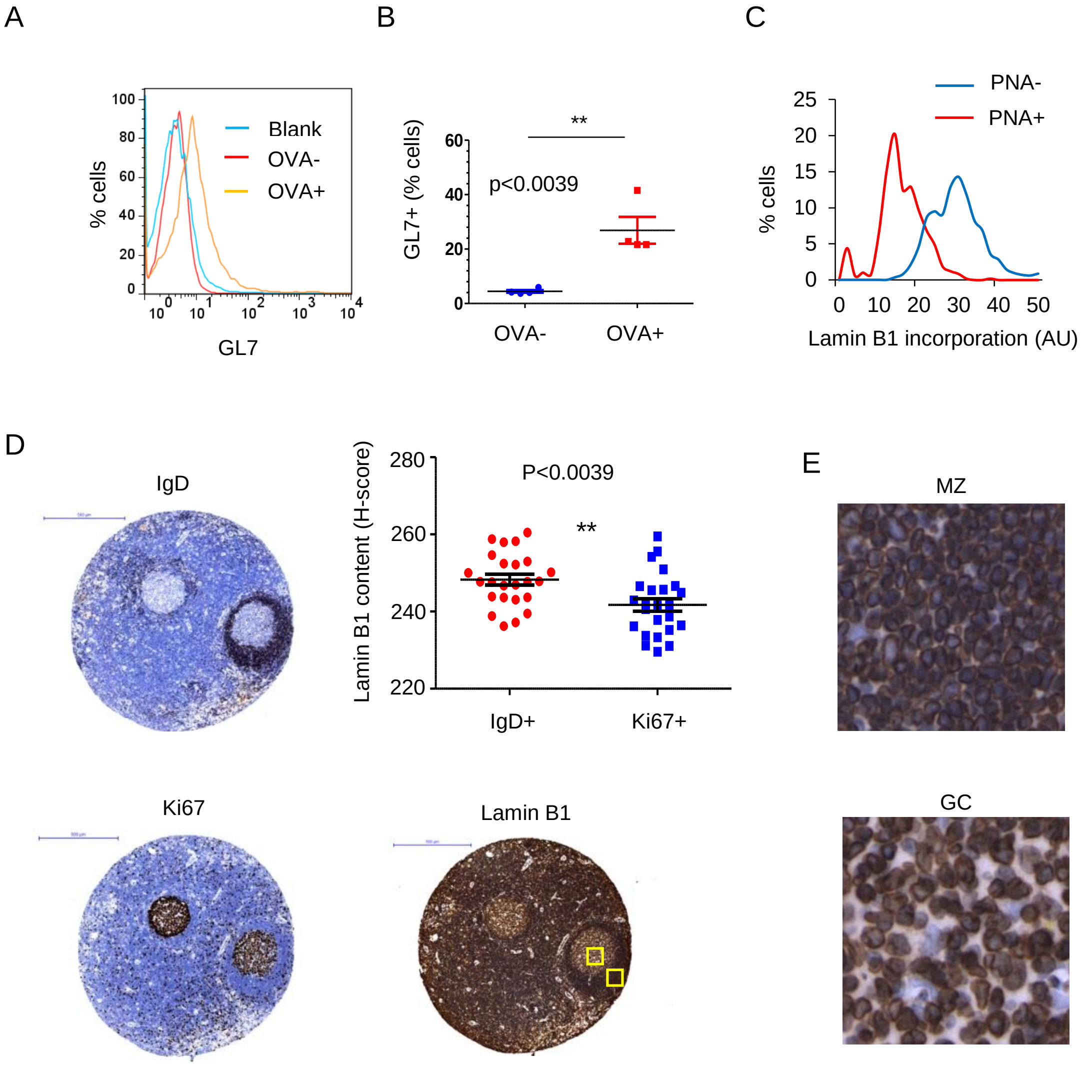

Supplementary figure 1. (A – B) FACS analysis of GL7 expression in control and OVA-immunised mouse splenocytes. n=4, p<0.005. (C) Absolute value redistribution of Lamin B1 incorporation in PNA+ vs PNA- splenocytes. (D) and (E): Immunohistochemical analysis of nuclear Lamin B1 levels in tissue microarrays of biopsies from 26 patients with previously diagnosed benign follicular hyperplasia. Lymphoid germinal centres were visualised as Ki67+ circular areas, whereas MZ B cells were identified by their IgD+ Ki67- phenotype. Lamin B1 levels on per cell basis were measured within the intra (Ki67+) or extra (IgD+) germinal areas using QuantiQuest module of the Pannoramic Viewer software. Lamin B1 content was expressed as H-score values representing the immunoreactivity of nuclear antigens (see Materials and Methods). At least three tissue core samples were analysed per patient, and average H-score values were calculated when more than one GC per core was observed. Bar 0.5 mm. (E) Higher magnification of a TMA core representing nuclear Lamin B1 immunopositivity of GC or MZ B cells within the yellow squares in (D).

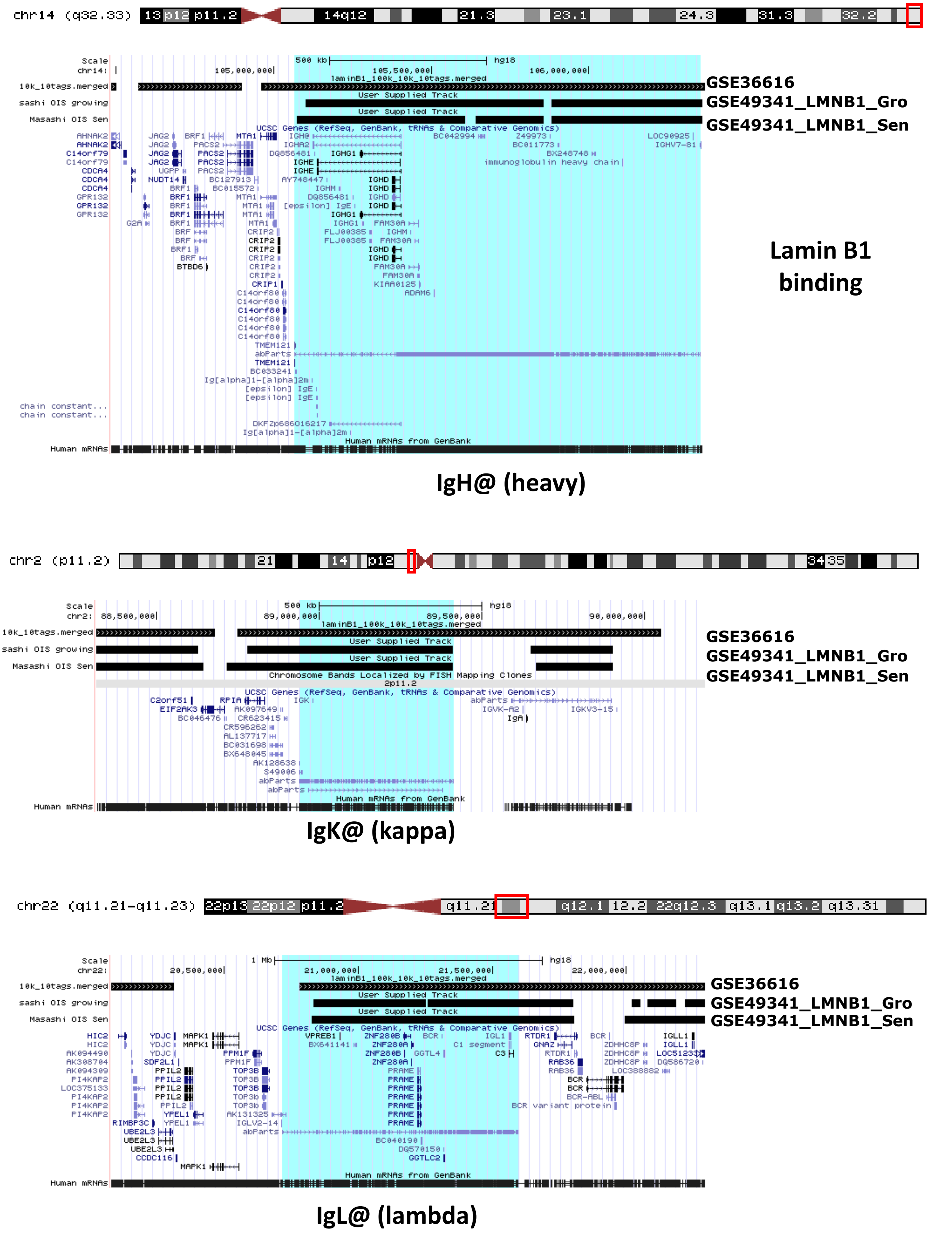

Supplementary figure 3. Heavy, kappa and lambda immunoglobulin domains co-localise with LADs. The topology of IgV domains (blue) was assessed in relation to previously published LAD calls (GSE36616: Shah et al.,. 2013, GSE49341, growing and senescent: Sadaie et al., 2013).

A

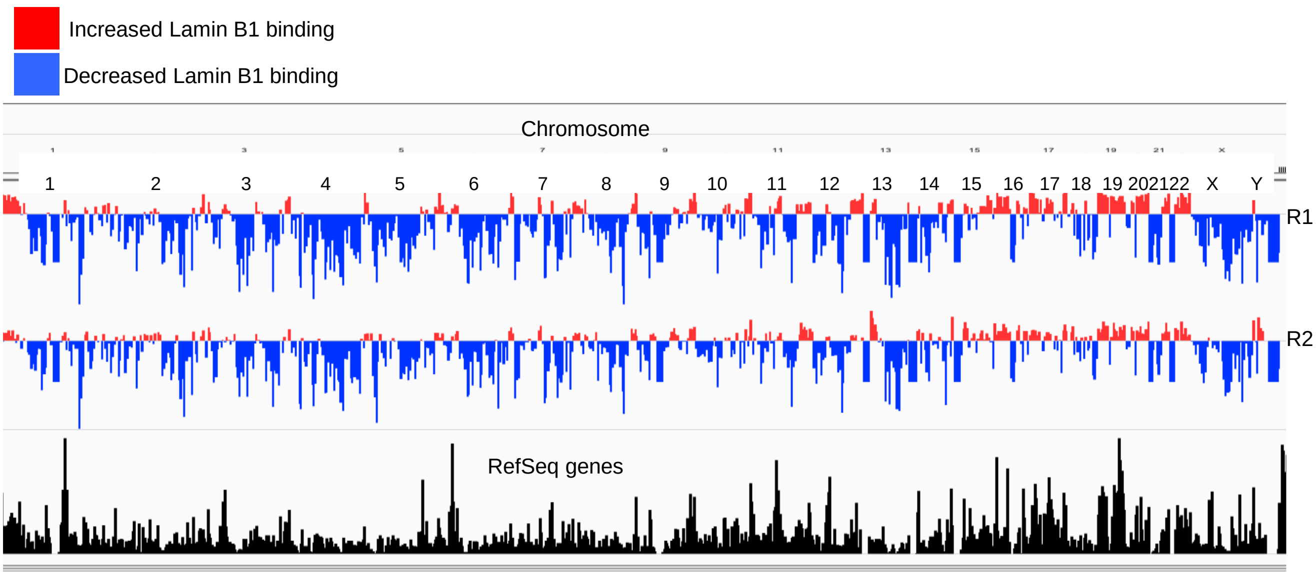

B

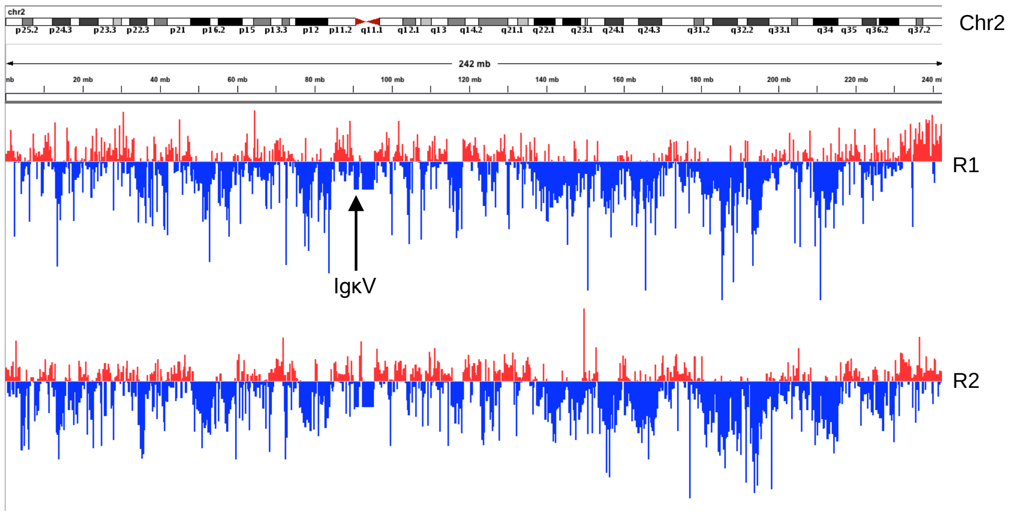

Supplementary figure 4. Lamin B1 genome binding dynamics in SHM uninduced vs. induced BL2 cells. SHM was induced in BL2 cells according to the protocol (see Materials and Methods) and ChIP-sequencing analysis of Lamin B1 genome binding was performed. (A) Global pan-genome distribution of Lamin B1 genome binding. With the exception of the smaller chromosomes 15-22, decreased Lamin B1 genome binding was an invariable consequence of BL2 cell activation. (B) Areas of decreased Lamin B1 binding coinciding with IgKV cluster.

A

V4-39-Jh5 Control

TTCTTCCTCCTGCTGGTGGCGGCTCCCAGATGTGAGTGTTTCTAGGATGCAGACATGGAGATATG
GGAGGCTGCCTCTGATCCCAGGGCTCACTGTGGGTTTTTCTGTTCACAGGGGTCCTGTCCCAGCT
GCAGCTGCAGGAGTCGGGCCCAGGACTGGTGAAGTCTTCGGAGaCCTTGTCCCTCACTTGCACT
GTCTCTGGTGGCTCCATCAGCAATACTAATTACTACTTGAGTTGGATCCGCCAGCCCCCAGGGAA
GGGGCTGGAATGGATTGGGACTATTTATTATAGTGGAATCACCTACTACAACCCATCCCTCGAGA
GTCGAGTCACCATGTCCGTAGACATGTCCAATAACCAATTCTCCCTGAAACTGAGTTCTGTGGCC
GCCGCAGACACGGCTGTGTATTACTGTGCGACCCACCAGTGGCTCGAAGGGGGGGgACTCTGGT
TCGACTCCTGGGGCCAGGGAACCCTGGTCACCGTCTCCTCAGGTGAGTCCTCACCACCCCCTCT
CTGAGTCCACTTAGGGAGACTCAACTTGCCAGGGTCTCGGGGTCAGAGTCTTGGAGGCATTTTG
GAGGTCAGGAAAGAAAGCCGGGGAG

B

V4-39-Jh5 SHM

TTCTTCCTCCTGCTGGTGGCGGCTCCCAGATGTGAGTGTTTCTAGGATGCAGACATGGAGATATG
GGAGGCTGCCTCTGATCCCAGGGCTCACTGTGGGTTTTTCTGTTCACAGGGGTCCTGTCCCAGCT
GCAGCTGCAGGAGTCGGGCCCAGGACTGGTGAAGTCTTCGGAGACCTTGTCCCTCACTTGCACT
GTCTCTGGTGGCTCCATCAGCAATACTAATTACTACTTGAGTTGGATCCGCCAGCCCCCAGGGAA
GGGGCTGGAATGGATTGGGACTATTTATTATAGTGGAATCACCTACTACAACCCATCCCTCGAGA
GTCGAGTCACCATGTCCGTAGACATGTCCAATAACCAATTCTCCCTGAAACTGAGTTCTGTGGCC
GCCGCAGACACGGCTCTGTATTACTGTGCGACCCACCAGTGCCTCGAAgGGGGGGGACTCTGGT
TCGACTCCTGGGGCCAGGGAACCCTGGTCACCGTCTCCTCAGGTGAGTCCTCACCACCCCCTCT
CTGAGTCCACTTAGGGAGACTCAACTTGCCAGGGTCTCGGGGTCAGAGTCTTGGAGGCATTTTG
GAGGTCAGGAAAGAAAGCCGGGGAG

C

Induced SHM (n=93)

	**A**	**T**	**G**	**C**	**Total**
**A**	-	1.07	2.15	1.07	
**T**	4.32	-	1.07	5.38	15.06
**G**	19.35	4.32	-	8.57	
**C**	7.53	32.25	12.9	-	84.92

D

LMNB1 RNAi (n=47)

	**A**	**T**	**G**	**C**	**Total**
**A**	-	2.12	8.5	4.26	
**T**	2.15	-	4.26	6.45	27.74
**G**	25.53	2.12	-	12.76	
**C**	4.26	21.27	6.45	-	72.39

Supplementary figure 4. (A) A reference sequence of the V4-39-Jh5 gene used for mutational analysis by high fidelity PCR, as described in the Materials and Methods. (B) Example sequence of V4-39-Jh5 following SHM induction in BL2 cells. (C) Sequence of V4-39-Jh5 following Lamin B1 siRNA knockdown as described in Materials and Methods. Green characters represent oligos binding site. (C) and (D) V4-39-Jh6 mutational pattern expressed as a percentage of nucleotide substitutions generated by IgM-CD19-CD21 cross-linking (C SHM-induced samples, 93 substitutions) or LMNB1 RNAi (D, 47 substitutions).

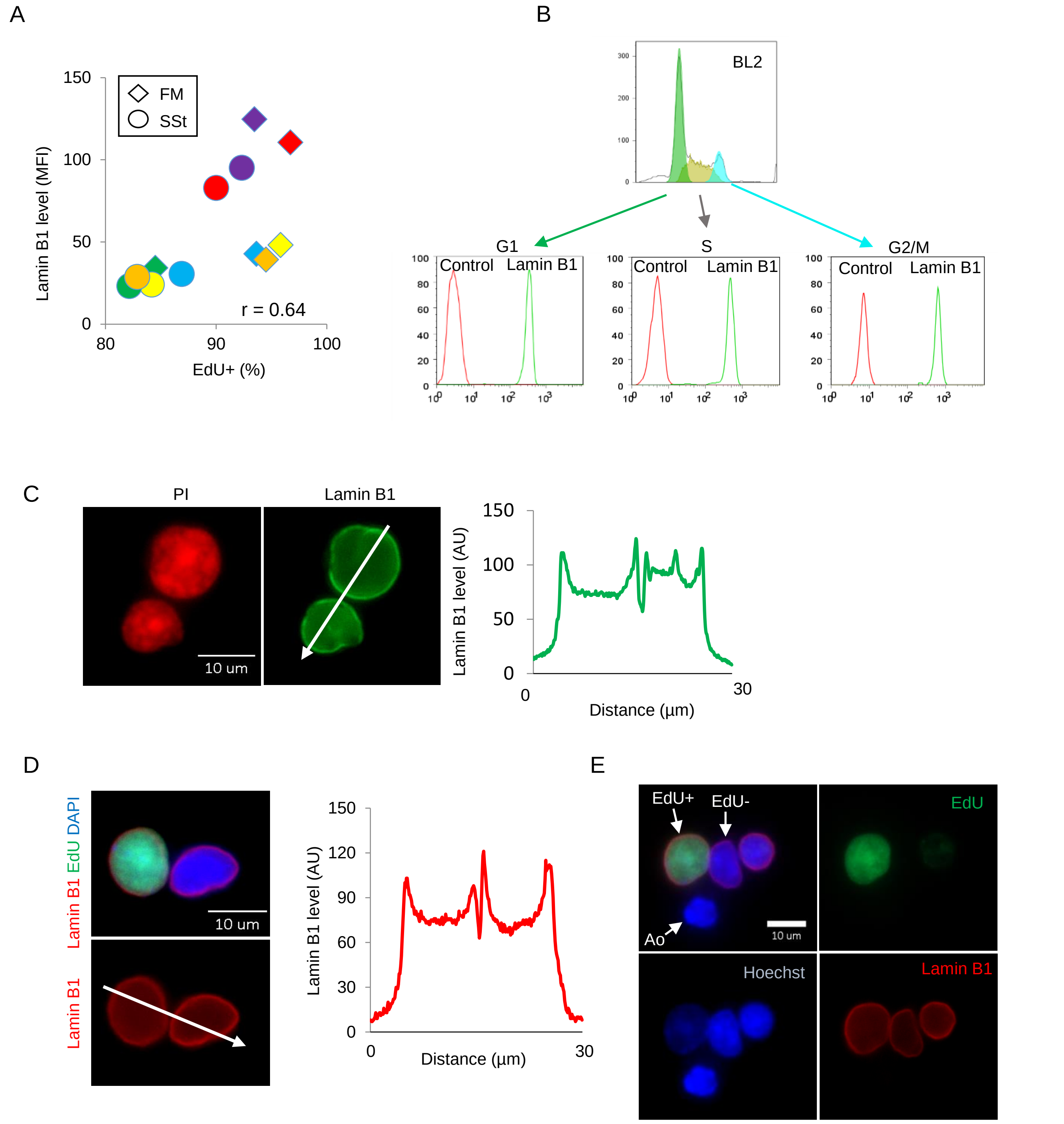

Supplementary figure 5 (A) Six cell lines (Pfeiffer, Raji, SUDHL4, EHEB, Karpas422 and BL2) were maintained either in full media (FM) or serum-free media (SSt). Cell proliferation assays revealed cells growing in full media proliferated more rapidly, and had a tendency to increase their Lamin B1 incorporation compared to steady-state cells. (B) FACS-mediated cell cycle analysis of Lamin B1 levels revealed there was a marginally higher amount of Lamin B1 of BL2 cells in G2 compared to cells in G1. (C) BL2 cells were processed for IF by staining with anti-Lamin B1 and counterstaining with propidium iodide (PI). Line scan analysis by MetaMorph revealed a marginally higher amount of nuclear Lamin B1 incorporation in cells in G2 phase. (D) and (E) EdU was added to BL2 cells and line scan analysis showed no difference of Lamin B1 incorporation between EdU+ and EdU- cells.

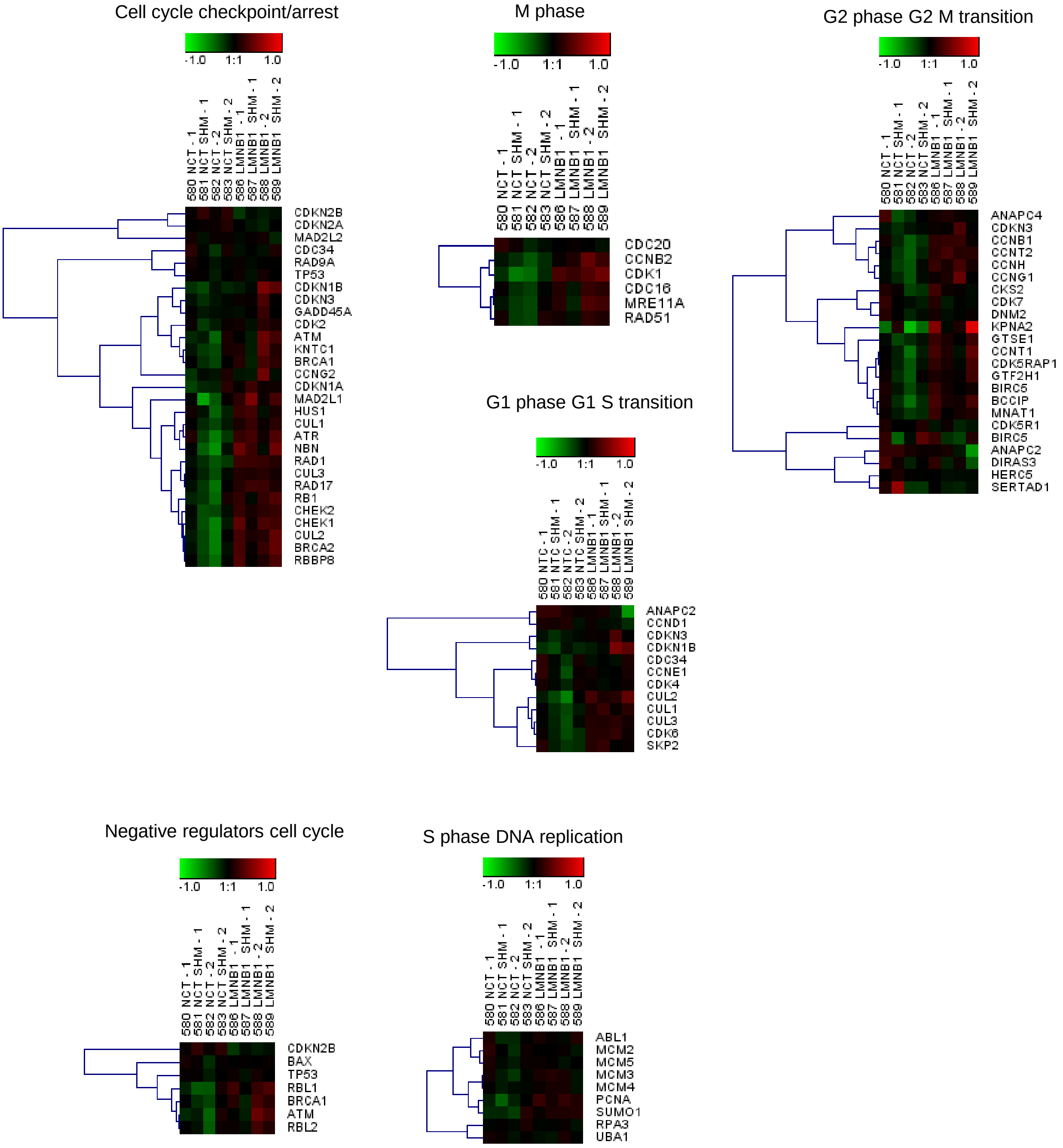

Supplementary figure 6. Expression profiles of cell-cycle regulatory genes in SHM-induced and/or LMNB1 siRNA treated BL2 cells. (NTC: control with non-targeting siRNA; NTC SHM: control with non-targeting siRNA after induction of SHM; LMNB1: LMNB1 RNAi transfected; LMNB1 SHM: LMNB1 RNAi transfected following induction of SHM. Experiments were conducted in two-independent BL2 cell passaged indicated with “-1” and “-2”. For siRNA-treated samples, a SMARTpool LMNB1 siRNA mixture of three siRNAs in equimolar concentrations was used. In samples where LMNB1 was reduced, there was a general upregulation of genes responsible for cell cycle checkpoint/arrest, as well as an upregulation of positive cell cycle regulatory genes. This occurred independently of SHM induction.

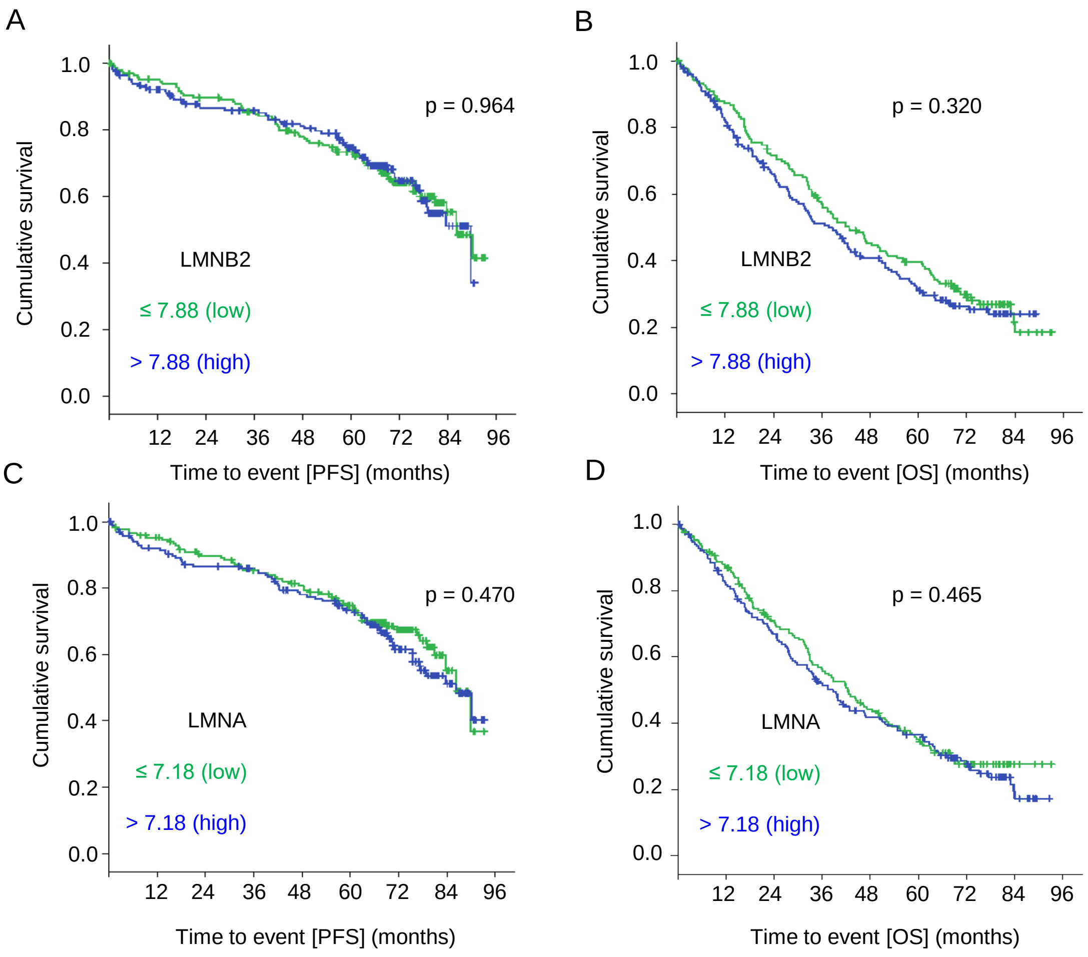

Supplementary figure 7. Kaplan-Meier estimates of Progression-free (PFS) and overall (OS) of CLL patients enrolled on the CLL8-trial as a factor of LMNB2 (A and B) or LMNA (C and D) expression.

A

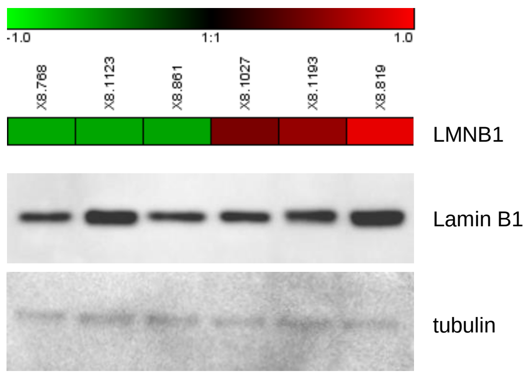

B

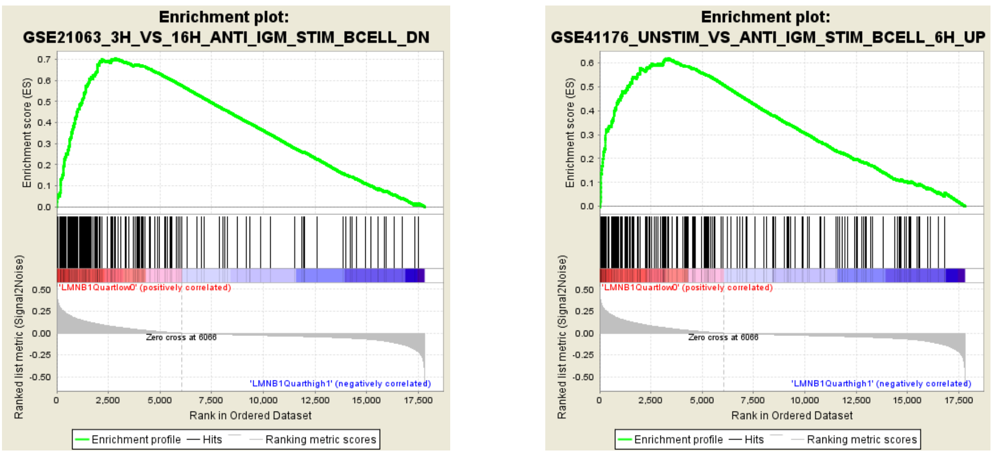

C

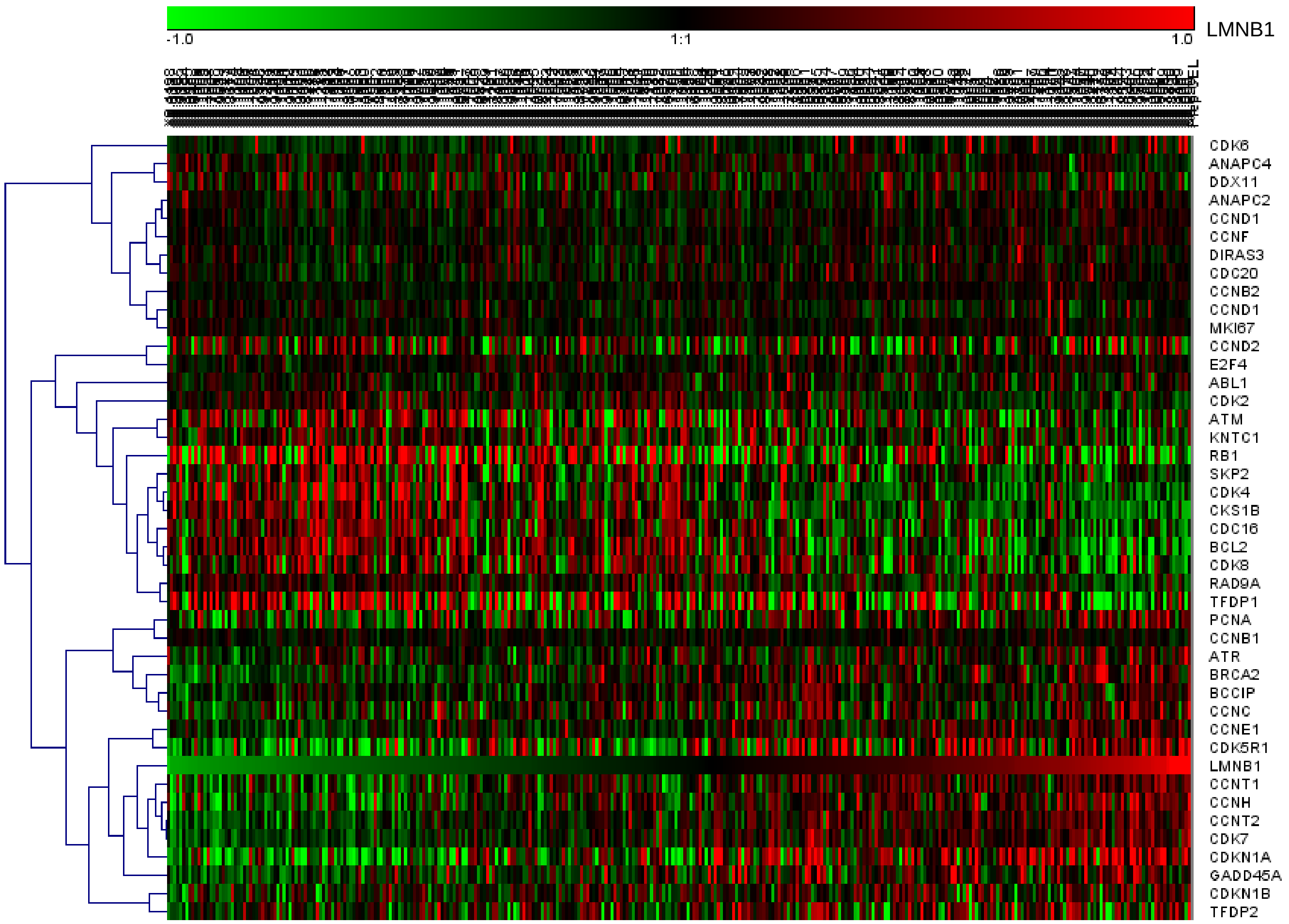

Supplementary figure 8. (A) Correlation of LMNB1 expression and Lamin B1 protein levels in six randomly selected CLL8 samples. (B) Gene Set Enrichment Analysis (GSEA) performed in CLL8 cohort revealed a strong association between low LMNB1 expression levels GEP phenotype of IgM stimulated B cells. (C) Gene expression profiles of cell cycle regulatory genes in CLL8 patient cohort.

A

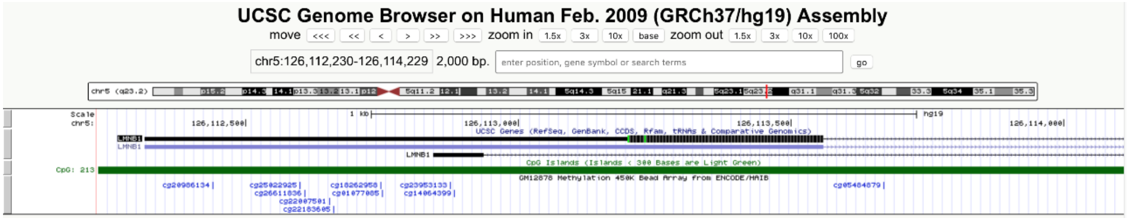

B

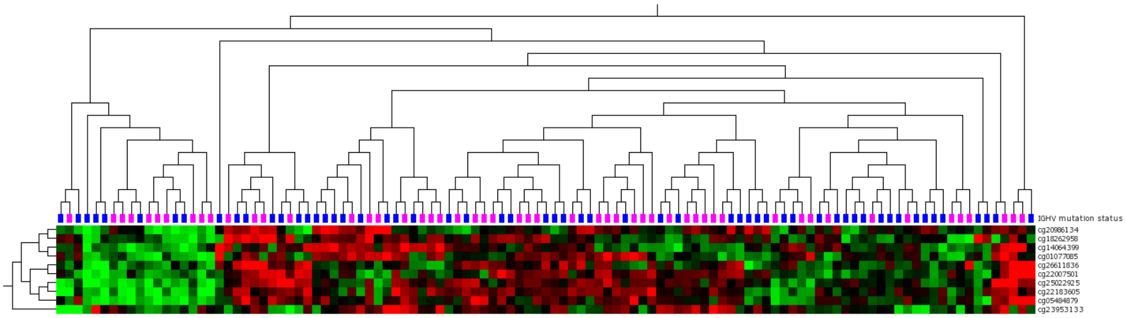

Pink - CLL patients with mutated (n=53) *IGHV* gene
Blue - CLL patients non-mutated (n=58) *IGHV* gene

C

Two-sample t test with equal variances

Group	Obs	Mean	Std. Err.	Std. Dev.	[95% Conf.	Interval]
0	58	.071024	.000932	.0070978	.0691578	.0728903
1	53	.0712772	.0011473	.0083528	.0689749	.0735795
combined	111	.0711449	.0007297	.0076878	.0696988	.072591
diff		-.0002532	.0014674		-.0031615	.0026551

diff = mean(0) - mean(1) t = -0.1726
Ho: diff = 0 degrees of freedom = 109

Ha: diff < 0	Ha: diff != 0	Ha: diff > 0
Pr(T < t) = 0.4317	Pr(|T| > |t|) = 0.8633	Pr(T > t) = 0.5683

Supplementary figure 9 (A) Promoter of *LMNB1* is contained within the CPG-island spanning chr5:126,112,230-126,114,229 (GRCh37/hg19 Assembly) and represented by ten probes on Infinium HumanMethylation450 Bead Chip. (B) To investigate the methylation status of *LMNB1* promoter in CLL, we extracted the methylation values for *LMNB1* promoter Infinium HumanMethylation450 Bead Chip probes and compared the methylation at those probes between CLL patients with mutated (n=53) and non-mutated (n=58) *IGHV* gene. (C) Two sample t-test with equal variances revealed no association between LMNB1 methylation and IGH mutational status. Infinium HumanMethylation450 BeadChip arrays data were obtained from CLL samples with more than 90% malignant cell content. The raw data were BMIQ normalised before comparison of methylation values

Human TaqMan probes
TaqMan Gene Expression Assay for LMNB1 human Hs01059210_m1<br>TaqMan Gene Expression Assay for 18S human, Hs99999901_s1<br>TaqMan Gene Expression Assay for ACTB human, Hs99999903_m1<br>TaqMan Gene Expression Assay for AICDA human Hs00757808_m1
Mouse Taqman Probes
TaqMan Gene Expression Assay for Mouse LMNB1 Mm00521949_m1<br>TaqMan Gene Expression Assay for Actb mouse Mm02619580_g1<br>TaqMan Gene Expression Assay for Rps18 mouse Mm02601777_g1<br>TaqMan Gene Expression Assay for mki67 mouse Mm01278617_m1<br>TaqMan Gene Expression Assay for Fas mouse Mm01204974_m1

Supplementary table 1. Taqman qRT-PCR probes used in this study

Baseline characteristics	Lamin B1 ≤ 6.51	Lamin B1 > 6.51	p value
Target analysis population, N	169	168	
Deletion in 11q, N (%)	169	166	
No	110 (65.1)	129 (77.7)	0.011
Yes	59 (34.9)	37 (22.3)	
Trisomy 12, N (%)	169	166	
No	156 (92.3)	141 (84.9)	0.033
Yes	13 (7.7)	25 (15.1)	
Type according to hierarchical model, N (%)	169	166	
17p deletion	18 (10.7)	10 (6.0)	0.021
11q deletion	55 (32.5)	35 (21.1)	
Trisomy 12	10 (5.9)	20 (12.0)	
No abnormalities	30 (17.8)	31 (18.7)	
13q deletion (single)	56 (33.1)	70 (42.2)	
IGHV mutational status, N (%)	165	162	
Unmutated	125 (75.8)	90 (55.6)	< 0.001
Mutated	40 (24.2)	72 (44.4)	

Supplementary table 2. Univariate comparison of CLL cytogenetic abnormalities in LMNB1 low (≤ 6.51) vs LMNB1 high (> 6.51) expressing samples.

CLL8 Lamin B1

Authors: Jasmin Bahlo
Sandra Robrecht
Version: 2.0

Content

Patient characteristics 2
Target analysis population 2
Patient characteristics of target analysis population 3
Overview on Lamin B1 5
Patient characteristics by Lamin B1 5
Efficacy 8
Progression free survival (PFS) by Lamin B1 8
PFS according to study treatment 10
PFS stratified comparisons 14
Survival 16
Overall survival (OS) by Lamin B1 16
OS according to study treatment 18
OS stratified comparisons 22
Appendix 24
LMNB2 24
Overview on LMNB2 24
Progression free survival (PFS) by LMNB2 25
Overall survival (OS) by LMNB2 27
LMNA 29
Overview on LMNA 29
Progression free survival (PFS) by LMNA 30
Overall survival (OS) by LMNB2 32

Patient characteristics

Target analysis population

Analysis population	FC	FCR	Total
**All patients (ITT), N**	**409**	**408**	**817**
Patients with missing Lamin B1, N (%)	240 (58.7)	240 (58.8)	480 (58.8)
**Target analysis population, N**	**169**	**168**	**337**

Patient characteristics of target analysis population

Baseline characteristics	FC	FCR	Total
**Target analysis population, N**	**169**	**168**	**337**
**Age at study entry (years)**	**169**	**168**	**337**
Median (range)	62 (36-81)	60 (35-77)	61 (35-81)
**Age group (years), N (%)**	**169**	**168**	**337**
≤ 60	77 (45.6)	85 (50.6)	162 (48.1)
> 60 & ≤ 65	47 (27.8)	45 (26.8)	92 (27.3)
> 65 & ≤ 70	31 (18.3)	28 (16.7)	59 (17.5)
> 70	14 (8.3)	10 (6.0)	24 (7.1)
**Gender, N (%)**	**169**	**168**	**337**
Female	41 (24.3)	40 (23.8)	81 (24.0)
Male	128 (75.7)	128 (76.2)	256 (76.0)
**Binet stage, N (%)**	**169**	**168**	**337**
A	10 (5.9)	11 (6.5)	21 (6.2)
B	107 (63.3)	99 (58.9)	206 (61.1)
C	52 (30.8)	58 (34.5)	110 (32.6)
**ECOG performance status, N (%)**	**159**	**163**	**322**
Median (range)	1 (0-1)	0 (0-2)	0 (0-2)
**Total CIRS score, N (%)**	**169**	**168**	**337**
Median (range)	2 (0-7)	1.5 (0-7)	2 (0-7)
**Deletion in 17p, N (%)**	**168**	**167**	**335**
No	153 (91.1)	154 (92.2)	307 (91.6)
Yes	15 (8.9)	13 (7.8)	28 (8.4)
**Deletion in 11q, N (%)**	**168**	**167**	**335**
No	125 (74.4)	114 (68.3)	239 (71.3)
Yes	43 (25.6)	53 (31.7)	96 (28.7)

**Trisomy 12, N (%)**	**168**	**167**	**335**
No	142 (84.5)	155 (92.8)	297 (88.7)
Yes	26 (15.5)	12 (7.2)	38 (11.3)
**Deletion in 13q, N (%)**	**168**	**167**	**335**
No	65 (38.7)	63 (37.7)	128 (38.2)
Yes	103 (61.3)	104 (62.3)	207 (61.8)
**Type according to hierarchical model, N (%)**	**168**	**167**	**335**
17p deletion	15 (8.9)	13 (7.8)	28 (8.4)
11q deletion	39 (23.2)	51 (30.5)	90 (26.9)
Trisomy 12	21 (12.5)	9 (5.4)	30 (9.0)
No abnormalities	30 (17.9)	31 (18.6)	61 (18.2)
13q deletion (single)	63 (37.5)	63 (37.7)	126 (37.6)
***IGHV* mutational status, N (%)**	**163**	**164**	**327**
Unmutated	106 (65.0)	109 (66.5)	215 (65.7)
Mutated	57 (35.0)	55 (33.5)	112 (34.3)
***TP53* mutational status, N (%)**	**167**	**164**	**331**
Unmutated	140 (83.8)	148 (90.2)	288 (87.0)
Mutated	27 (16.2)	16 (9.8)	43 (13.0)
**Serum thymidine kinase (U/L)**	**148**	**158**	**306**
Median (range)	23.4 (3.5-855.0)	17.1 (2.7-970.0)	20.1 (2.7-970.0)
**Serum thymidine kinase (U/L), N (%)**	**148**	**158**	**306**
≤ 10.0	26 (17.6)	38 (24.1)	64 (20.9)
> 10.0	122 (82.4)	120 (75.9)	242 (79.1)
**Serum β_2-microglobulin (mg/l)**	**148**	**158**	**306**
Median (range)	2.9 (1.1-9.2)	2.7 (0.9-8.0)	2.8 (0.9-9.2)
**Serum β_2-microglobulin (mg/l) N (%)**	**148**	**158**	**306**
≤ 3.5	99 (66.9)	110 (69.6)	209 (68.3)
> 3.5	49 (33.1)	48 (30.4)	97 (31.7)

Overview on Lamin B1

Characteristic	FC	FCR	Total
**Target analysis population, N**	**169**	**168**	**337**
**Lamin B1**			
Mean	6.57	6.60	6.58
25% percentile	6.24	6.28	6.25
**Median (range)**	**6.50 (5.76-8.05)**	**6.52 (5.90-7.84)**	**6.51 (5.76-8.05)**
75% percentile	6.82	6.91	6.87

Patient characteristics by Lamin B1

Baseline characteristics	Lamin B1 ≤ 6.51	Lamin B1 > 6.51	p value
**Target analysis population, N**	**169**	**168**	
**Age at study entry (years)**	**169**	**168**	
Median (range)	62 (36-78)	60 (35-81)	0.392
**Age group (years), N (%)**	**169**	**168**	
≤ 60	76 (45.0)	86 (51.2)	0.507
> 60 & ≤ 65	48 (28.4)	44 (26.2)	
> 65 & ≤ 70	34 (20.1)	25 (14.9)	
> 70	11 (6.5)	13 (7.7)	
**Gender, N (%)**	**169**	**168**	
Female	40 (23.7)	41 (24.4)	0.874
Male	129 (76.3)	127 (75.6)	
**Binet stage, N (%)**	**169**	**168**	
A	8 (4.7)	13 (7.7)	0.469
B	103 (60.9)	103 (61.3)	
C	58 (34.3)	52 (31.0)	
**ECOG performance status, N (%)**	**163**	**159**	
Median (range)	0 (0-2)	1 (0-1)	0.544

**Total CIRS score, N (%)**	**169**	**168**	
Median (range)	1 (0-7)	2 (0-7)	0.320
**Deletion in 17p, N (%)**	**169**	**166**	
No	151 (89.3)	156 (94.0)	0.126
Yes	18 (10.7)	10 (6.0)	
**Deletion in 11q, N (%)**	**169**	**166**	
No	110 (65.1)	129 (77.7)	0.011
Yes	59 (34.9)	37 (22.3)	
**Trisomy 12, N (%)**	**169**	**166**	
No	156 (92.3)	141 (84.9)	0.033
Yes	13 (7.7)	25 (15.1)	
**Deletion in 13q, N (%)**	**169**	**166**	
No	63 (37.3)	65 (39.2)	0.723
Yes	106 (62.7)	101 (60.8)	
**Type according to hierarchical model, N (%)**	**169**	**166**	
17p deletion	18 (10.7)	10 (6.0)	0.021
11q deletion	55 (32.5)	35 (21.1)	
Trisomy 12	10 (5.9)	20 (12.0)	
No abnormalities	30 (17.8)	31 (18.7)	
13q deletion (single)	56 (33.1)	70 (42.2)	
***IGHV* mutational status, N (%)**	**165**	**162**	
Unmutated	125 (75.8)	90 (55.6)	< 0.001
Mutated	40 (24.2)	72 (44.4)	
***TP53* mutational status, N (%)**	**167**	**164**	
Unmutated	141 (84.4)	147 (89.6)	0.159
Mutated	26 (15.6)	17 (10.4)	

**Serum thymidine kinase (U/L)**	**157**	**149**	
Median (range)	23.5 (2.7-970.0)	16.7 (2.8-855.0)	0.010
**Serum thymidine kinase (U/L), N (%)**	**157**	**149**	
≤ 10.0	30 (19.1)	34 (22.8)	0.425
> 10.0	127 (80.9)	115 (77.2)	
**Serum β_2-microglobulin (mg/l)**	**157**	**149**	
Median (range)	3.0 (1.3-9.2)	2.6 (0.9-8.0)	0.021
**Serum β_2-microglobulin (mg/l) N (%)**	**157**	**149**	
≤ 3.5	102 (65.0)	107 (71.8)	0.198
> 3.5	55 (35.0)	42 (28.2)	

Efficacy

Progression free survival (PFS) by Lamin B1

PFS	Pts, N	Events, N	Median months	2-year Survival, %	3-year Survival, %	5-year Survival, %	7-year Survival, %
**Lamin B1**	**337**	**235**	**39.9**				
**≤ 6.51 (low)**	169	124	32.4	61.2	45.6	30.1	18.8
**> 6.51 (high)**	168	111	49.9	76.7	62.9	41.4	22.2

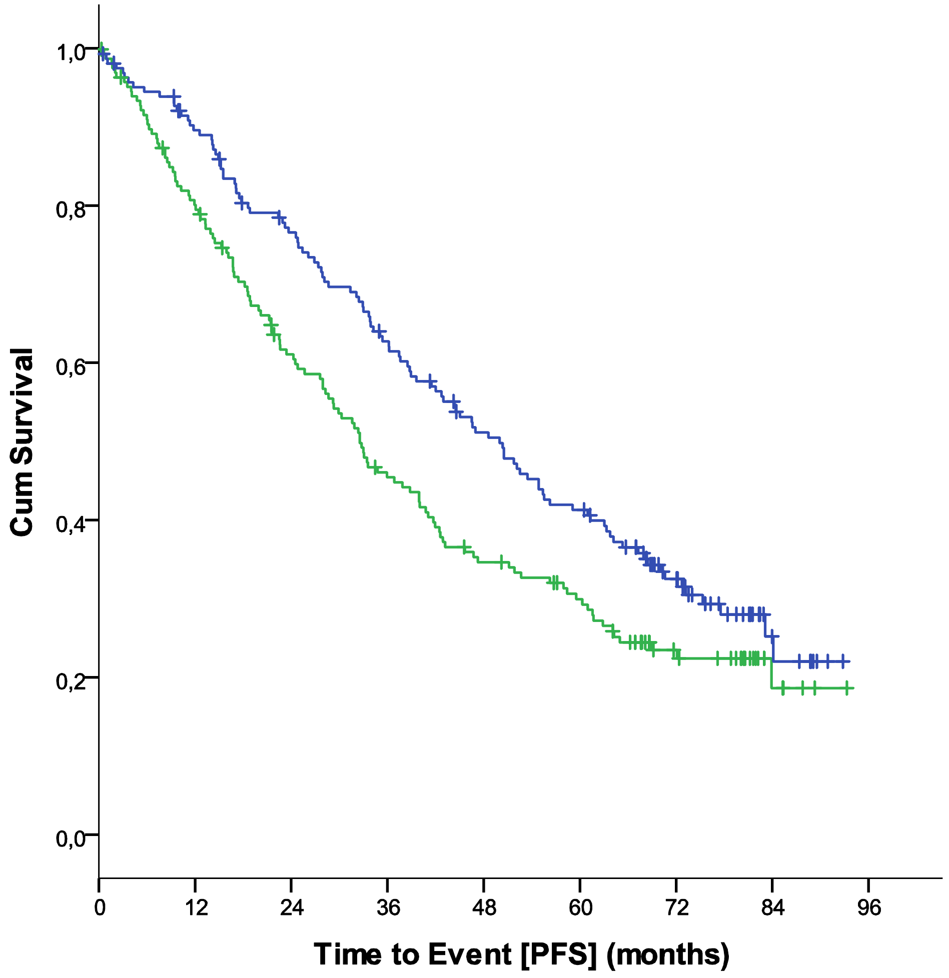

p = 0.010 (log-rank)

COX regression PFS	Univariate comparison	Hazard ratio [HR]	95% Confidence Interval		*p* value
			Lower	Upper	
**Lamin B1**					
High	***vs.*** low	0.715	0.553	0.924	0.010

PFS	Pts, N	Events, N	Median months	2-year Survival, %	3-year Survival, %	5-year Survival, %	7-year Survival, %
**Lamin B1**	**337**	**235**	**39.9**				
≤ 6.25	84	65	31.7	58.6	40.8	26.4	13.0
> 6.25 & ≤ 6.51	85	59	38.7	63.8	50.3	33.9	25.8
> 6.51 & ≤ 6.87	84	54	50.2	79.2	63.1	38.3	31.5
> 6.87	84	57	46.8	74.2	62.7	44.6	19.5

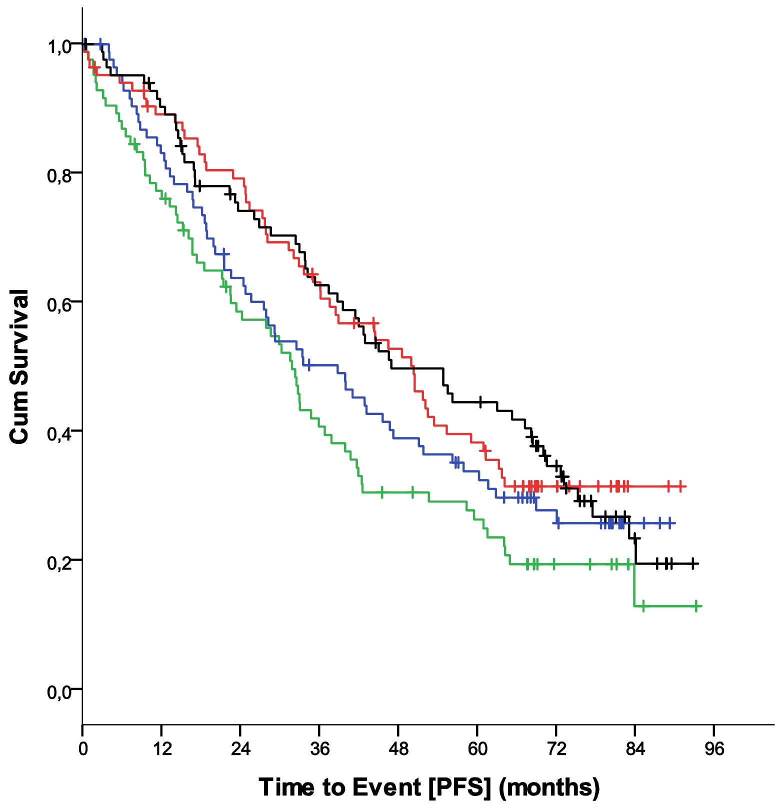

p = 0.026 (log-rank)

COX regression PFS	Univariate comparison	Hazard ratio [HR]	95% Confidence Interval		*p* value
			Lower	Upper	
**Lamin B1**					
> 6.25 & ≤ 6.51	***vs.*** ≤ 6.25	0.767	0.539	1.092	0.141
> 6.51 & ≤ 6.87	***vs.*** ≤ 6.25	0.626	0.436	0.898	0.011
> 6.87	***vs.*** ≤ 6.25	0.623	0.436	0.890	0.009

COX regression PFS	Univariate comparison	Hazard ratio [HR]	95% Confidence Interval		*p* value
			Lower	Upper	
**Lamin B1 (continuous variable)**		0.665	0.483	0.916	0.013

PFS according to study treatment

Study treatment = FC

PFS	Pts, N	Events, N	Median months	2-year Survival, %	3-year Survival, %	5-year Survival, %	7-year Survival, %
**Lamin B1**	**169**	**125**	**31.7**				
**≤ 6.51 (low)**	87	68	24.3	51.8	30.3	19.6	15.1
**> 6.51 (high)**	82	57	37.3	69.0	52.4	29.5	19.9

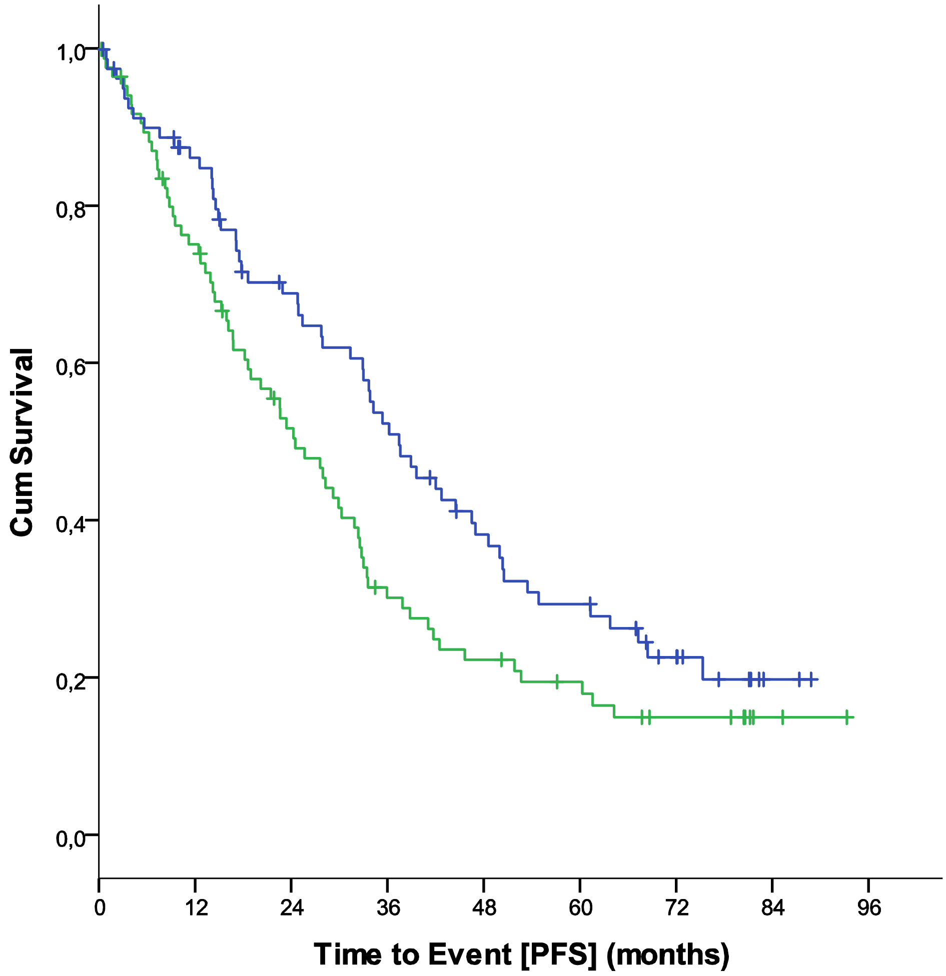

p = 0.031 (log-rank)

COX regression PFS	Univariate comparison	Hazard ratio [HR]	95% Confidence Interval		*p* value
			Lower	Upper	
**Lamin B1**					
High	***vs.*** low	0.680	0.477	0.968	0.033

PFS	Pts, N	Events, N	Median months	2-year Survival, %	3-year Survival, %	5-year Survival, %	7-year Survival, %
**Lamin B1**	**169**	**125**	**31.7**				
**≤ 6.25**	48	37	24.1	52.3	30.9	21.1	15.8
**> 6.25 & ≤ 6.51**	39	31	24.3	51.4	29.7	17.8	14.3[1]
**> 6.51 & ≤ 6.87**	44	31	37.5	73.8	54.1	28.6	22.8[2]
**> 6.87**	38	26	37.3	63.8	51.0	31.3	19.5

[1] At time point of last observation (month 81.5)
[2] At time point of last observation (month 82.8)

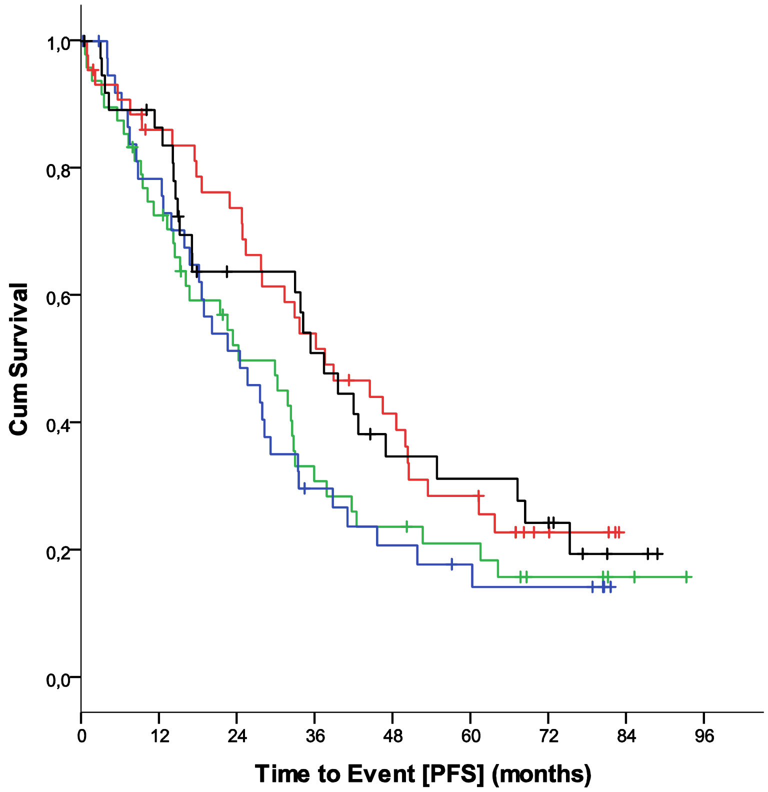

p = 0.195 (log-rank)

COX regression PFS	Univariate comparison	Hazard ratio [HR]	95% Confidence Interval		*p* value
			Lower	Upper	
**Lamin B1**					
> 6.25 & ≤ 6.51	***vs.*** ≤ 6.25	1.051	0.652	1.695	0.837
> 6.51 & ≤ 6.87	***vs.*** ≤ 6.25	0.681	0.422	1.100	0.116
> 6.87	***vs.*** ≤ 6.25	0.713	0.431	1.178	0.187

COX regression PFS	Univariate comparison	Hazard ratio [HR]	95% Confidence Interval		*p* value
			Lower	Upper	
**Lamin B1 (continuous variable)**		0.658	0.426	1.019	0.060

Study treatment = FCR

PFS	Pts, N	Events, N	Median months	2-year Survival, %	3-year Survival, %	5-year Survival, %	7-year Survival, %
**Lamin B1**	**168**	**110**	**56.1**				
≤ 6.51 (low)	82	56	43.1	70.7	60.8	40.6	22.3
> 6.51 (high)	86	54	62.9	83.7	72.1	51.7	25.7

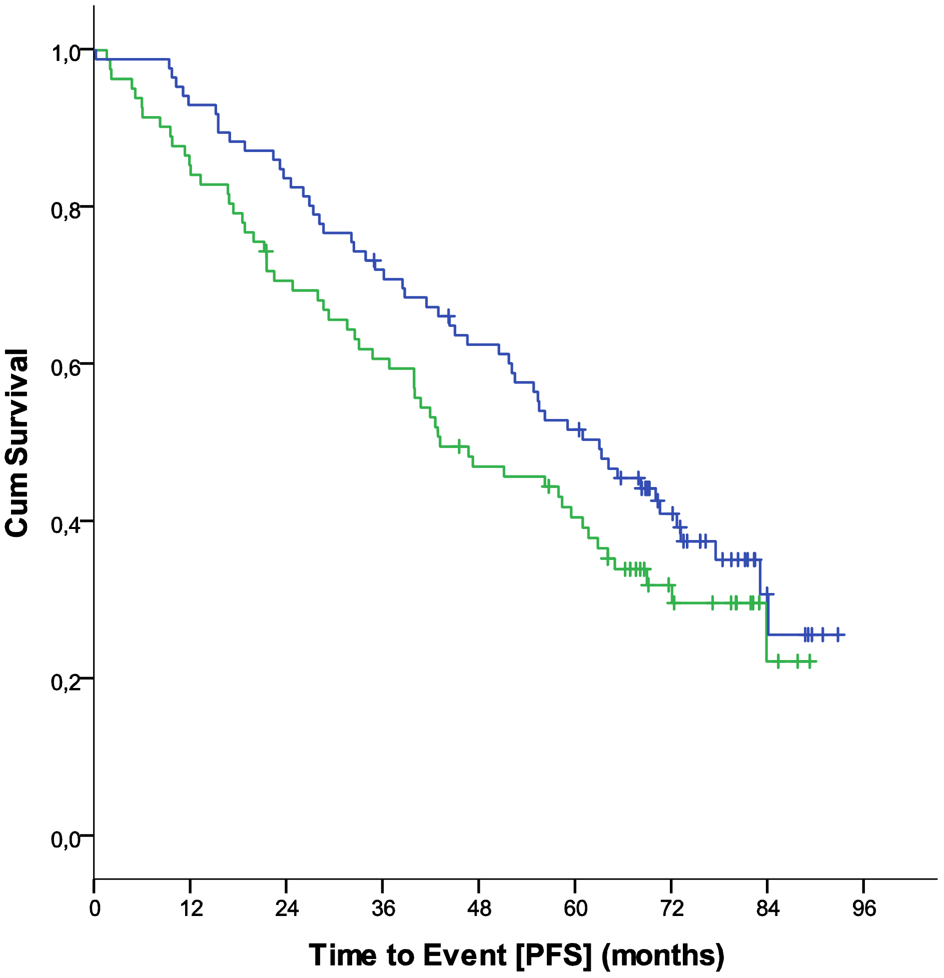

p = 0.119 (log-rank)

COX regression PFS	Univariate comparison	Hazard ratio [HR]	95% Confidence Interval		*p* value
			Lower	Upper	
**Lamin B1**					
High	***vs.*** low	0.743	0.511	1.081	0.121

PFS	Pts, N	Events, N	Median months	2-year Survival, %	3-year Survival, %	5-year Survival, %	7-year Survival, %
**Lamin B1**	**168**	**110**	**56.1**				
**≤ 6.25**	36	28	36.7	66.7	52.8	32.9	0.0[1]
**> 6.25 & ≤ 6.51**	46	28	56.1	73.8	67.1	46.9	34.5
**> 6.51 & ≤ 6.87**	40	23	59.0	85.0	72.4	48.4	40.3
**> 6.87**	46	31	65.2	82.6	71.7	54.3	20.2

[1] At time point of last observation (month 83.7)

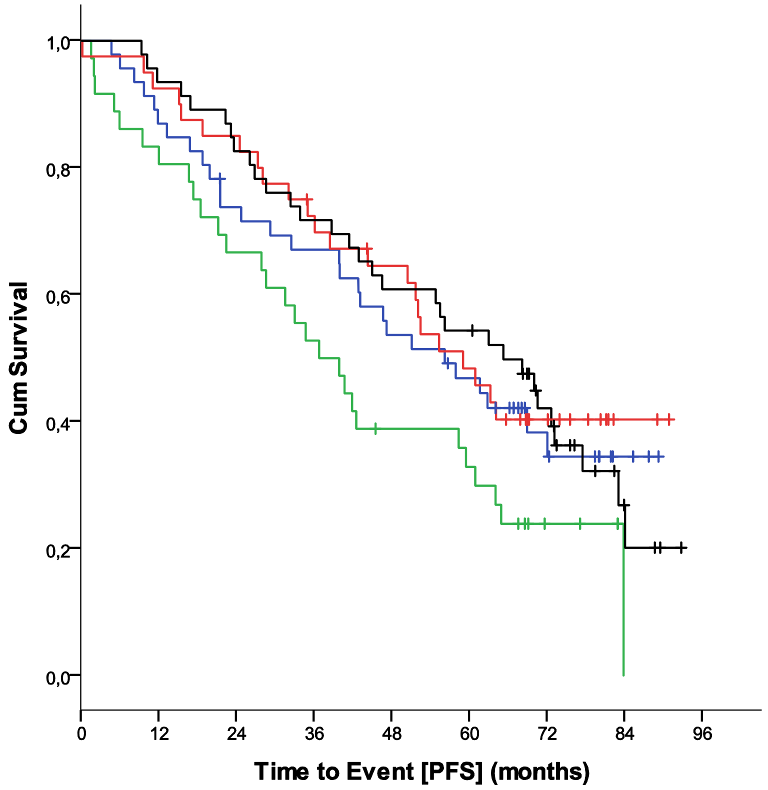

p = 0.103 (log-rank)

COX regression PFS	Univariate comparison	Hazard ratio [HR]	95% Confidence Interval		*p* value
			Lower	Upper	
**Lamin B1**					
> 6.25 & ≤ 6.51	***vs.*** ≤ 6.25	0.623	0.368	1.053	0.077
> 6.51 & ≤ 6.87	***vs.*** ≤ 6.25	0.552	0.317	0.959	0.035
> 6.87	***vs.*** ≤ 6.25	0.584	0.349	0.977	0.041

COX regression PFS	Univariate comparison	Hazard ratio [HR]	95% Confidence Interval		*p* value
			Lower	Upper	
**Lamin B1 (continuous variable)**		0.686	0.429	1.098	0.116

PFS stratified comparisons

Method: enter

COX regression PFS	Univariate comparison	Hazard ratio [HR]	95% Confidence Interval		*p* value
			Lower	Upper	
**Lamin B1**					
High	***vs.*** low	0.714	0.552	0.923	0.010
**Study treatment**					
FCR	***vs.*** FC	0.560	0.432	0.725	< 0.001

COX regression PFS	Univariate comparison	Hazard ratio [HR]	95% Confidence Interval		*p* value
			Lower	Upper	
**Lamin B1**					
High	***vs.*** low	0.780	0.598	1.017	0.067
**Study treatment**					
FCR	***vs.*** FC	0.563	0.434	0.731	< 0.001
***IGHV* mutational status**					
Unmutated	***vs.*** mutated	1.878	1.388	2.542	< 0.001

COX regression PFS	Univariate comparison	Hazard ratio [HR]	95% Confidence Interval		*p* value
			Lower	Upper	
**Lamin B1**					
High	***vs.*** low	0.815	0.624	1.066	0.136
**Study treatment**					
FCR	***vs.*** FC	0.517	0.396	0.674	< 0.001
***IGHV* mutational status**					
Unmutated	***vs.*** mutated	1.704	1.252	2.319	0.001
**Deletion in 17p**					
Yes	***vs.*** no	5.543	3.530	8.702	< 0.001

COX regression PFS	Univariate comparison	Hazard ratio [HR]	95% Confidence Interval		*p* value
			Lower	Upper	
**Lamin B1 (continuous variable)**		0.677	0.492	0.931	0.017
**Study treatment**					
FCR	***vs.*** FC	0.564	0.435	0.731	< 0.001

COX regression PFS	Univariate comparison	Hazard ratio [HR]	95% Confidence Interval		*p* value
			Lower	Upper	
**Lamin B1 (continuous variable)**		0.783	0.569	1.079	0.135
**Study treatment**					
FCR	***vs.*** FC	0.567	0.437	0.737	< 0.001
***IGHV* mutational status**					
Unmutated	***vs.*** mutated	1.892	1.397	2.563	< 0.001

COX regression PFS	Univariate comparison	Hazard ratio [HR]	95% Confidence Interval		*p* value
			Lower	Upper	
**Lamin B1 (continuous variable)**		0.736	0.539	1.006	0.054
**Study treatment**					
FCR	***vs.*** FC	0.525	0.402	0.684	< 0.001
***IGHV* mutational status**					
Unmutated	***vs.*** mutated	1.676	1.231	2.282	0.001
**Deletion in 17p**					
Yes	***vs.*** no	5.976	3.794	9.413	< 0.001

Survival

Overall survival (OS) by Lamin B1

OS	Pts, N	Events, N	Median months	2-year Survival, %	3-year Survival, %	5-year Survival, %	7-year Survival, %
**Lamin B1**	**337**	**121**	**86.3**				
≤ 6.51 (low)	169	74	83.6	84.2	81.7	68.3	43.1
> 6.51 (high)	168	47	NR	92.1	89.5	79.7	63.2

NR, not reached

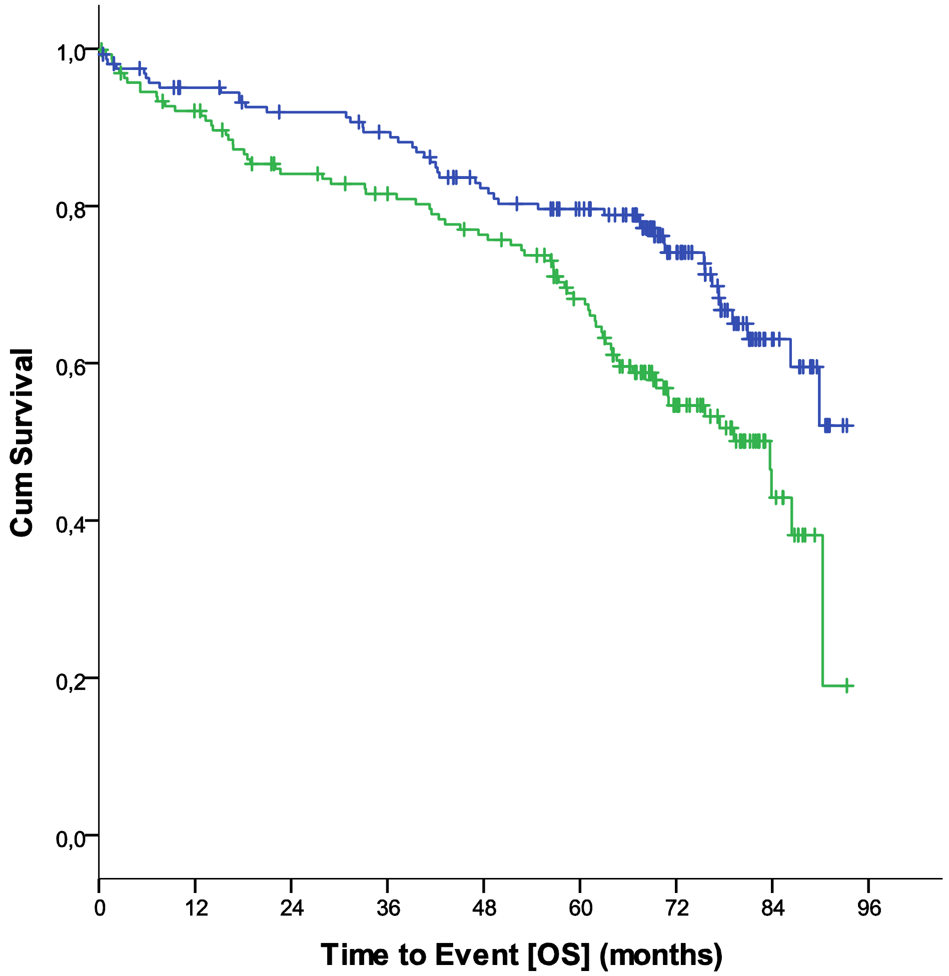

p = 0.001 (log-rank)

COX regression OS	Univariate comparison	Hazard ratio [HR]	95% Confidence Interval		*p* value
			Lower	Upper	
**Lamin B1**					
High	*vs.* low	0.551	0.381	0.795	0.001

OS	Pts, N	Events, N	Median months	2-year Survival, %	3-year Survival, %	5-year Survival, %	7-year Survival, %
**Lamin B1**	**337**	**121**	**86.3**				
**≤ 6.25**	84	38	79.1	83.2	81.9	65.4	41.2
**> 6.25 & ≤ 6.51**	85	36	83.6	85.3	81.6	71.2	44.9
**> 6.51 & ≤ 6.87**	84	23	89.7	90.3	86.6	79.0	67.3
**> 6.87**	84	24	NR	93.8	92.5	80.4	60.2

NR, not reached

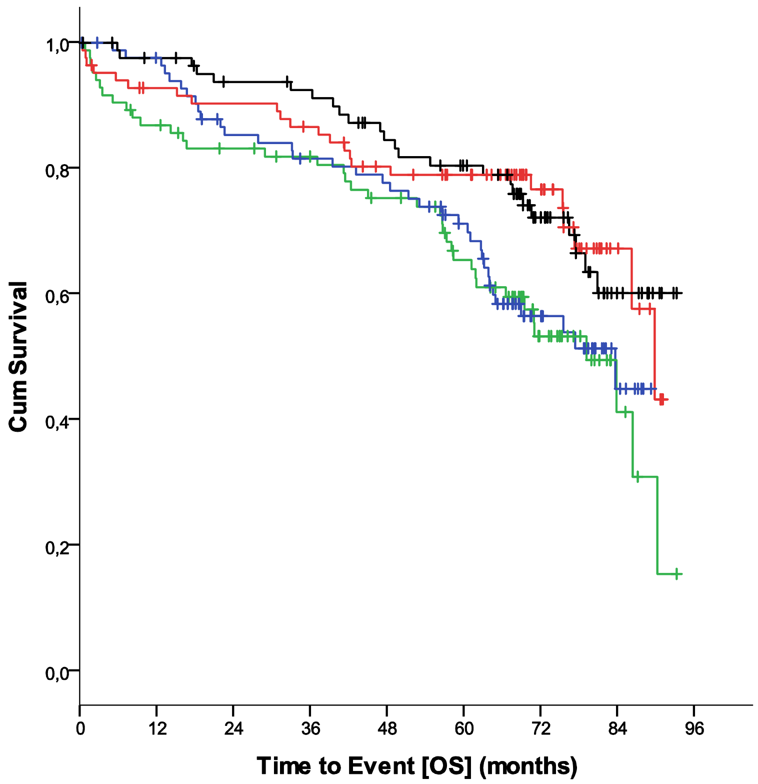

p = 0.013 (log-rank)

COX regression OS	Univariate comparison	Hazard ratio [HR]	95% Confidence Interval		*p* value
			Lower	Upper	
**Lamin B1**					
> 6.25 & ≤ 6.51	***vs.*** ≤ 6.25	0.892	0.565	1.408	0.622
> 6.51 & ≤ 6.87	***vs.*** ≤ 6.25	0.529	0.315	0.888	0.016
> 6.87	***vs.*** ≤ 6.25	0.512	0.307	0.855	0.010

COX regression OS	Univariate comparison	Hazard ratio [HR]	95% Confidence Interval		*p* value
			Lower	Upper	
**Lamin B1 (continuous variable)**		0.557	0.353	0.879	0.012

OS according to study treatment

Study treatment = FC

OS	Pts, N	Events, N	Median months	2-year Survival, %	3-year Survival, %	5-year Survival, %	7-year Survival, %
**Lamin B1**	**169**	**68**	**78.9**				
**≤ 6.51 (low)**	87	42	69.4	80.8	78.3	62.1	35.3
**> 6.51 (high)**	82	26	89.7	88.5	84.4	73.0	54.7

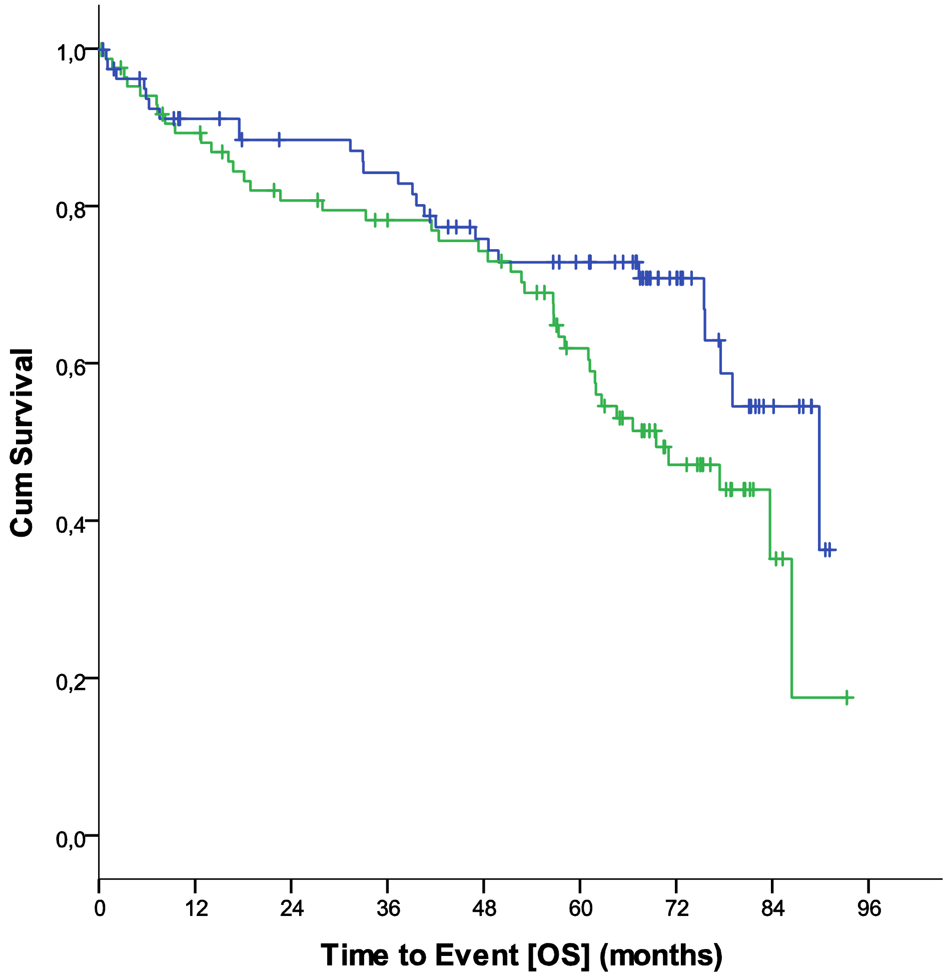

p = 0.039 (log-rank)

COX regression OS	Univariate comparison	Hazard ratio [HR]	95% Confidence Interval		*p* value
			Lower	Upper	
**Lamin B1**					
High	*vs.* low	0.599	0.366	0.980	0.042

OS	Pts, N	Events, N	Median months	2-year Survival, %	3-year Survival, %	5-year Survival, %	7-year Survival, %
**Lamin B1**	**169**	**68**	**78.9**				
≤ 6.25	48	22	69.4	83.1	83.1	65.0	45.9
> 6.25 & ≤ 6.51	39	20	64.5	78.4	73.0	58.8	21.9
> 6.51 & ≤ 6.87	44	14	89.7	86.0	81.1	73.6	55.2
> 6.87	38	12	NR	91.5	88.3	72.0	53.2

NR, not reached

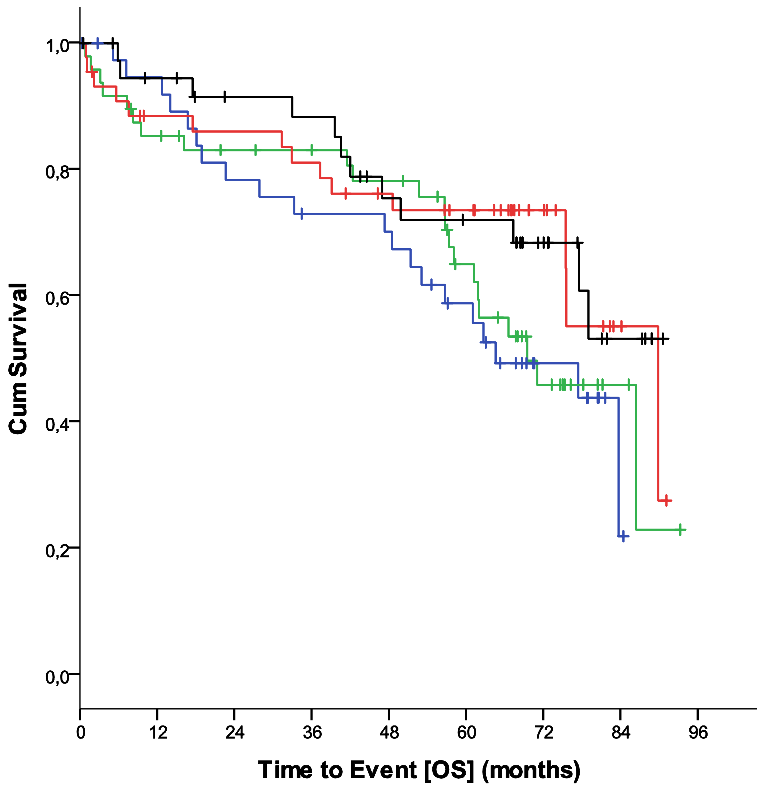

p = 0.215 (log-rank)

COX regression OS	Univariate comparison	Hazard ratio [HR]	95% Confidence Interval		*p* value
			Lower	Upper	
**Lamin B1**					
> 6.25 & ≤ 6.51	***vs.*** ≤ 6.25	1.133	0.617	2.081	0.687
> 6.51 & ≤ 6.87	***vs.*** ≤ 6.25	0.657	0.336	1.285	0.220
> 6.87	***vs.*** ≤ 6.25	0.610	0.301	1.237	0.170

COX regression OS	Univariate comparison	Hazard ratio [HR]	95% Confidence Interval		*p* value
			Lower	Upper	
**Lamin B1 (continuous variable)**		0.682	0.380	1.224	0.199

Study treatment = FCR

OS	Pts, N	Events, N	Median months	2-year Survival, %	3-year Survival, %	5-year Survival, %	7-year Survival, %
**Lamin B1**	**168**	**53**	**90.2**				
≤ 6.51 (low)	82	32	90.2	87.7	85.1	74.6	50.4
> 6.51 (high)	86	21	NR	95.3	94.2	85.7	69.2

NR, not reached

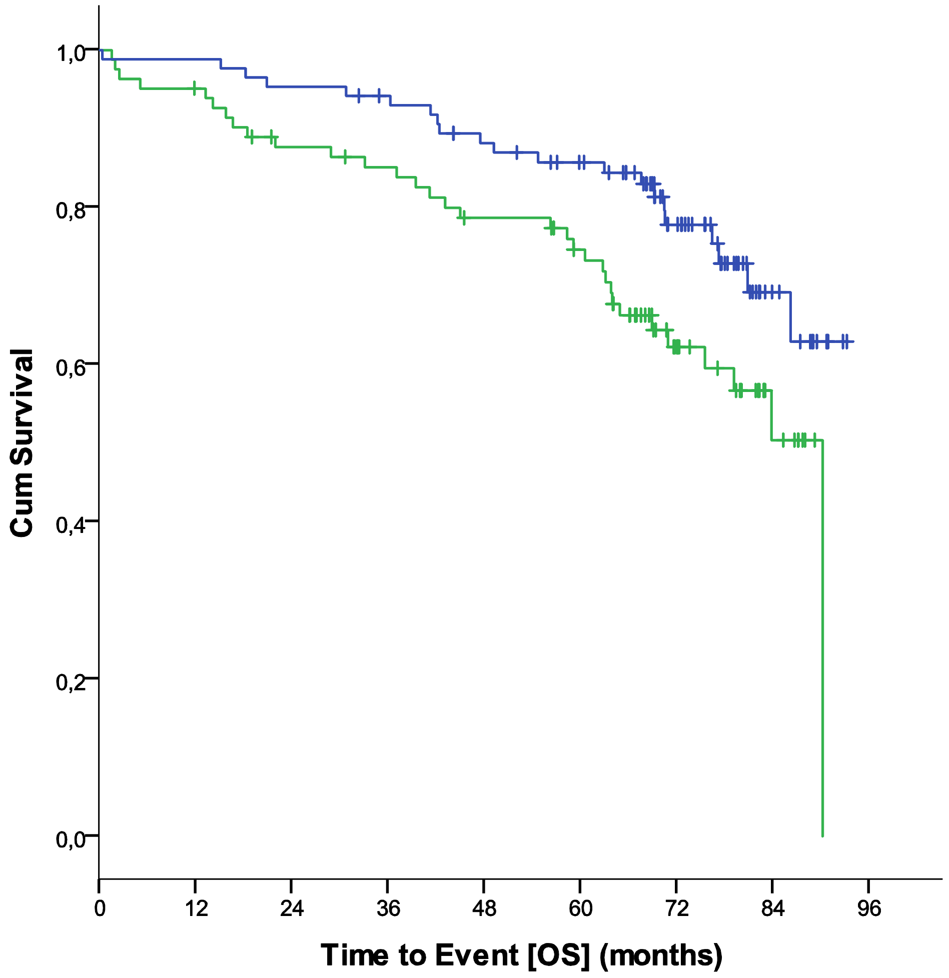

p = 0.018 (log-rank)

COX regression OS	Univariate comparison	Hazard ratio [HR]	95% Confidence Interval		*p* value
			Lower	Upper	
**Lamin B1**					
High	*vs.* low	0.518	0.298	0.900	0.020

OS	Pts, N	Events, N	Median months	2-year Survival, %	3-year Survival, %	5-year Survival, %	7-year Survival, %
**Lamin B1**	**168**	**53**	**90.2**				
≤ 6.25	36	16	83.7	83.3	80.6	65.9	36.3
> 6.25 & ≤ 6.51	46	16	NR	91.0	88.7	81.5	57.7
> 6.51 & ≤ 6.87	40	9	NR	95.0	92.5	84.8	75.5
> 6.87	46	12	NR	95.7	95.7	86.6	64.7

NR, not reached

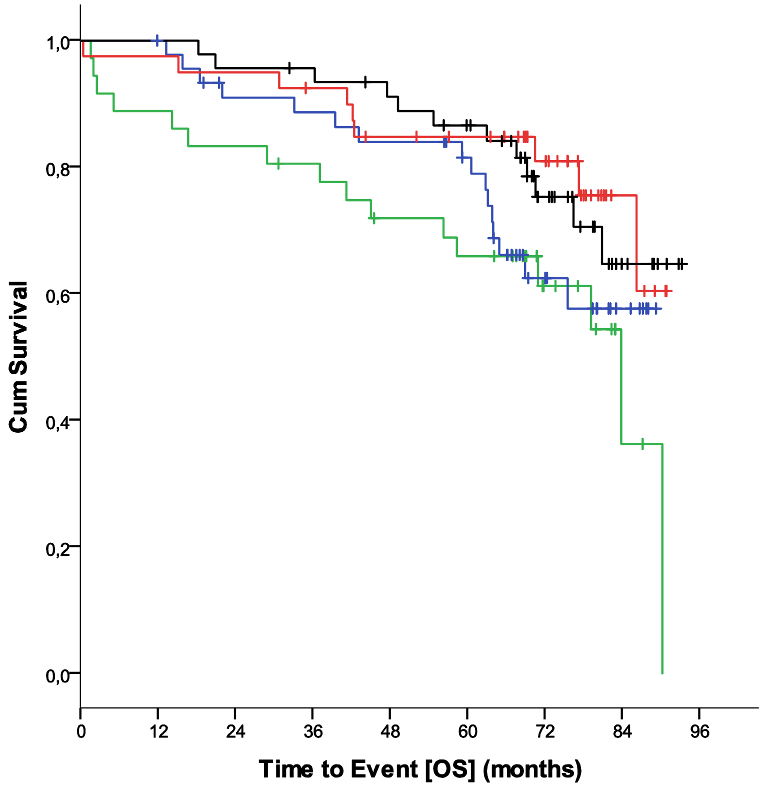

p = 0.086 (log-rank)

COX regression OS	Univariate comparison	Hazard ratio [HR]	95% Confidence Interval		*p* value
			Lower	Upper	
**Lamin B1**					
> 6.25 & ≤ 6.51	*vs.* ≤ 6.25	0.749	0.374	1.501	0.415
> 6.51 & ≤ 6.87	*vs.* ≤ 6.25	0.418	0.185	0.946	0.036
> 6.87	*vs.* ≤ 6.25	0.466	0.220	0.986	0.046

COX regression OS	Univariate comparison	Hazard ratio [HR]	95% Confidence Interval		*p* value
			Lower	Upper	
**Lamin B1 (continuous variable)**		0.459	0.223	0.942	0.034

OS stratified comparisons

Method: enter

COX regression OS	Univariate comparison	Hazard ratio [HR]	95% Confidence Interval		*p* value
			Lower	Upper	
**Lamin B1**					
High	***vs.*** low	0.562	0.389	0.811	0.002
**Study treatment**					
FCR	***vs.*** FC	0.619	0.431	0.888	0.009

COX regression OS	Univariate comparison	Hazard ratio [HR]	95% Confidence Interval		*p* value
			Lower	Upper	
**Lamin B1**					
High	***vs.*** low	0.641	0.439	0.934	0.021
**Study treatment**					
FCR	***vs.*** FC	0.630	0.438	0.906	0.013
***IGHV* mutational status**					
Unmutated	***vs.*** mutated	2.580	1.598	4.167	< 0.001

COX regression OS	Univariate comparison	Hazard ratio [HR]	95% Confidence Interval		*p* value
			Lower	Upper	
**Lamin B1**					
High	***vs.*** low	0.735	0.499	1.082	0.119
**Study treatment**					
FCR	***vs.*** FC	0.652	0.453	0.939	0.021
***IGHV* mutational status**					
Unmutated	***vs.*** mutated	2.204	1.350	3.599	0.002
**Deletion in 17p**					
Yes	***vs.*** no	5.304	3.245	8.670	< 0.001

COX regression OS	Univariate comparison	Hazard ratio [HR]	95% Confidence Interval		p value
			Lower	Upper	
**Lamin B1 (continuous variable)**		0.579	0.368	0.911	0.018
**Study treatment**					
FCR	***vs.*** FC	0.620	0.432	0.890	0.010

COX regression OS	Univariate comparison	Hazard ratio [HR]	95% Confidence Interval		p value
			Lower	Upper	
**Lamin B1 (continuous variable)**		0.712	0.452	1.120	0.142
**Study treatment**					
FCR	***vs.*** FC	0.631	0.438	0.909	0.013
***IGHV* mutational status**					
Unmutated	***vs.*** mutated	2.662	1.647	4.304	< 0.001

COX regression OS	Univariate comparison	Hazard ratio [HR]	95% Confidence Interval		p value
			Lower	Upper	
**Lamin B1 (continuous variable)**		0.793	0.509	1.234	0.303
**Study treatment**					
FCR	***vs.*** FC	0.655	0.453	0.945	0.024
***IGHV* mutational status**					
Unmutated	***vs.*** mutated	2.254	1.380	3.679	0.001
**Deletion in 17p**					
Yes	***vs.*** no	5.557	3.414	9.046	< 0.001

Appendix

LMNB2

Overview on LMNB2

Characteristic	FC	FCR	Total
**Target analysis population, N**	**169**	**168**	**337**
**LMNB2**			
Mean	7.90	7.86	7.88
25% percentile	7.76	7.73	7.75
**Median (range)**	**7.90 (7.37-8.49)**	**7.86 (7.29-8.52)**	**7.88 (7.29-8.52)**
75% percentile	8.02	7.98	8.02

Progression free survival (PFS) by LMNB2

COX regression PFS	Univariate comparison	Hazard ratio [HR]	95% Confidence Interval		*p* value
			Lower	Upper	
**LMNB2 (continuous variable)**		1.866	0.988	3.524	0.054
**Study treatment**					
FCR	***vs.*** FC	0.569	0.439	0.737	< 0.001

PFS	Pts, N	Events, N	Median months	2-year Survival, %	3-year Survival, %	5-year Survival, %	7-year Survival, %
**LMNB2**	**337**	**235**	**39.9**				
**≤ 7.88 (low)**	169	119	42.8	71.7	57.1	39.6	18.4
**> 7.88 (high)**	168	116	37.7	66.1	51.2	31.7	24.0

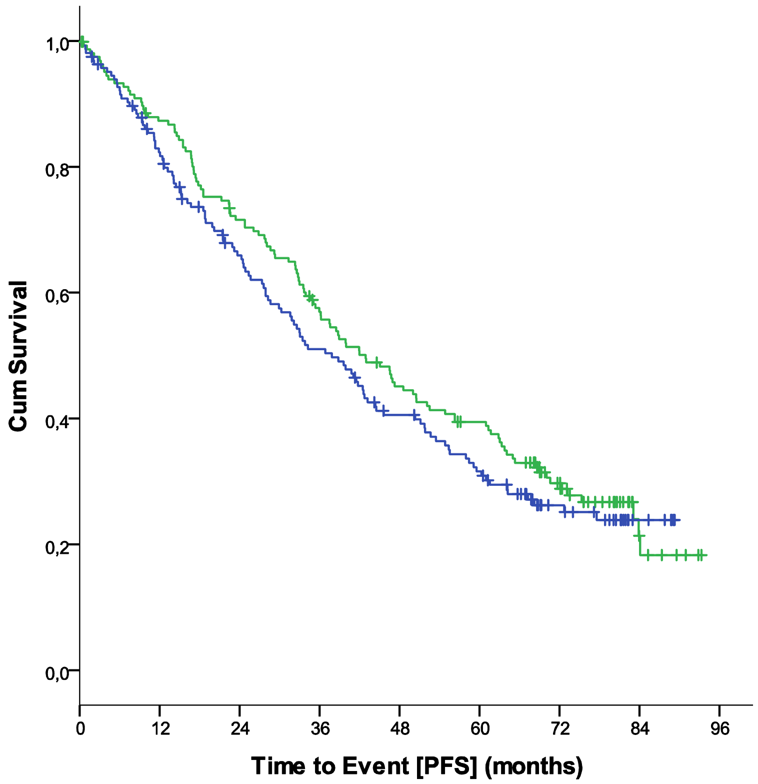

p = 0.320 (log-rank)

COX regression PFS	Univariate comparison	Hazard ratio [HR]	95% Confidence Interval		*p* value
			Lower	Upper	
**LMNB2**					
High	***vs.*** low	1.139	0.881	1.471	0.320

PFS	Pts, N	Events, N	Median months	2-year Survival, %	3-year Survival, %	5-year Survival, %	7-year Survival, %
**LMNB2**	**337**	**235**	**39.9**				
≤ 7.75	84	55	46.8	73.0	60.5	42.6	22.2
> 7.75 & ≤ 7.88	85	64	39.8	70.6	53.8	36.7	24.1
> 7.88 & ≤ 8.02	84	59	41.6	68.6	57.6	32.0	25.7
> 8.02	84	57	30.1	63.4	44.1	31.4	21.7

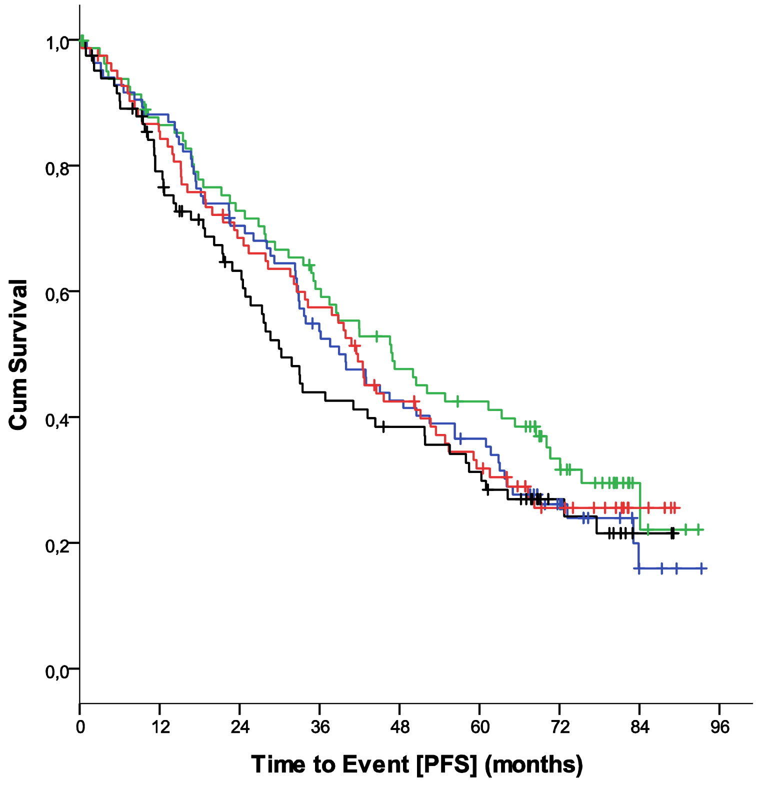

p = 0.436 (log-rank)

COX regression PFS	Univariate comparison	Hazard ratio [HR]	95% Confidence Interval		*p* value
			Lower	Upper	
**LMNB2**					
> 7.75 & ≤ 7.88	***vs.*** ≤ 7.75	1.208	0.842	1.732	0.305
> 7.88 & ≤ 8.02	***vs.*** ≤ 7.75	1.167	0.808	1.685	0.411
> 8.02	***vs.*** ≤ 7.75	1.362	0.940	1.974	0.103

Overall survival (OS) by LMNB2

COX regression OS	Univariate comparison	Hazard ratio [HR]	95% Confidence Interval		p value
			Lower	Upper	
**LMNB2 (continuous variable)**		1.456	0.585	3.622	0.420
**Study treatment**					
FCR	*vs.* FC	0.616	0.428	0.885	0.009

OS	Pts, N	Events, N	Median months	2-year Survival, %	3-year Survival, %	5-year Survival, %	7-year Survival, %
**LMNB2**	**337**	**121**	**86.3**				
≤ 7.88 (low)	169	63	83.6	86.3	85.4	73.4	55.4
> 7.88 (high)	168	58	NR	89.7	85.8	74.7	51.1

NR, not reached

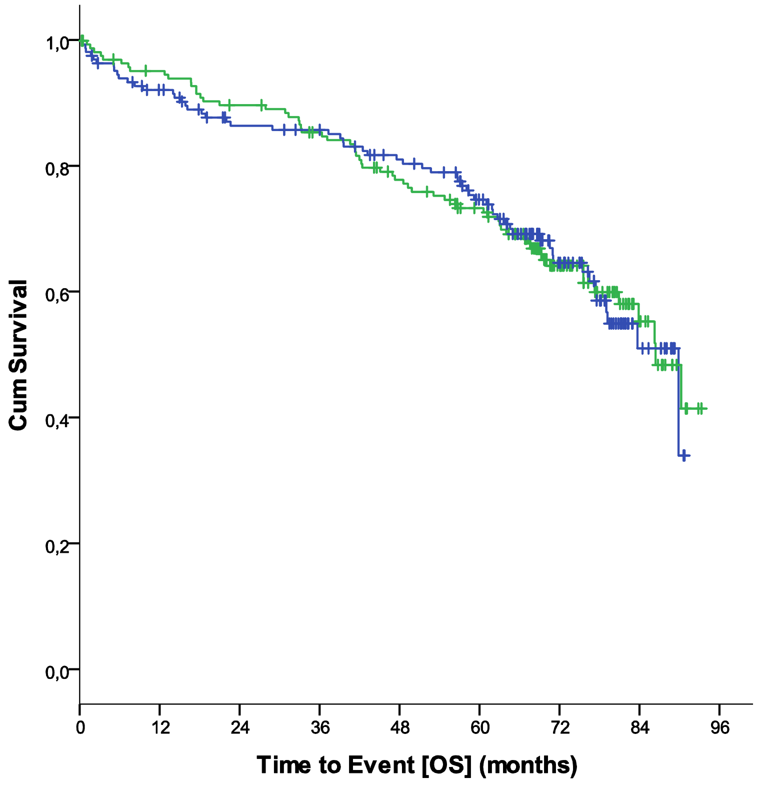

p = 0.964 (log-rank)

COX regression OS	Univariate comparison	Hazard ratio [HR]	95% Confidence Interval		p value
			Lower	Upper	
**LMNB2**					
High	*vs.* low	1.008	0.705	1.442	0.964

OS	Pts, N	Events, N	Median months	2-year Survival, %	3-year Survival, %	5-year Survival, %	7-year Survival, %
**LMNB2**	**337**	**121**	**86.3**				
≤ 7.75	84	27	NR	91.3	87.5	74.4	57.8
> 7.75 & ≤ 7.88	85	36	86.1	88.2	83.5	72.4	53.5
> 7.88 & ≤ 8.02	84	26	89.7	85.4	85.4	80.2	59.4
> 8.02	84	32	83.3	87.6	86.2	68.7	37.6

NR, not reached

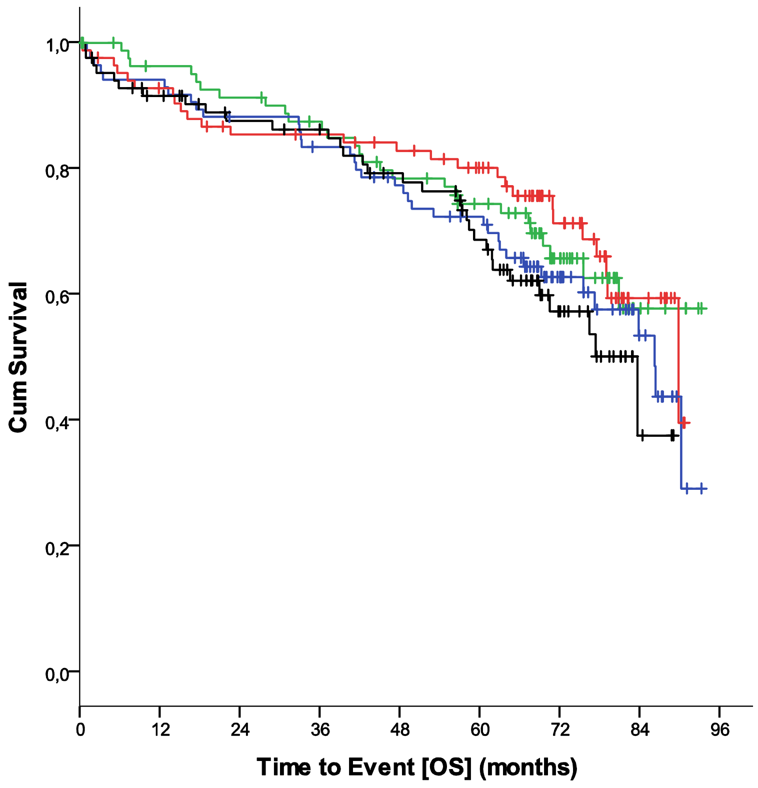

p = 0.339 (log-rank)

COX regression OS	Univariate comparison	Hazard ratio [HR]	95% Confidence Interval		*p* value
			Lower	Upper	
**LMNB2**					
> 7.75 & ≤ 7.88	***vs.*** ≤ 7.75	1.263	0.766	2.082	0.360
> 7.88 & ≤ 8.02	***vs.*** ≤ 7.75	0.931	0.543	1.596	0.794
> 8.02	***vs.*** ≤ 7.75	1.411	0.844	2.361	0.189

LMNA

Overview on LMNA

Characteristic	FC	FCR	Total
**Target analysis population, N**	**169**	**168**	**337**
**LMNA**			
Mean	7.26	7.27	7.26
25% percentile	6.89	6.90	6.89
**Median (range)**	**7.16 (6.43-9.85)**	**7.16 (6.38-9.27)**	**7.16 (6.38-9.85)**
75% percentile	7.49	7.53	7.51

Progression free survival (PFS) by LMNA

COX regression PFS	Univariate comparison	Hazard ratio [HR]	95% Confidence Interval		*p* value
			Lower	Upper	
**LMNA (continuous variable)**		0.997	0.807	1.233	0.980
**Study treatment**					
FCR	*vs.* FC	0.560	0.432	0.725	< 0.001

PFS	Pts, N	Events, N	Median months	2-year Survival, %	3-year Survival, %	5-year Survival, %	7-year Survival, %
**LMNA**	**337**	**235**	**39.9**				
≤ 7.16 (low)	169	114	42.3	70.9	56.3	35.1	27.6
> 7.16 (high)	168	121	38.6	66.9	52.0	36.5	17.2

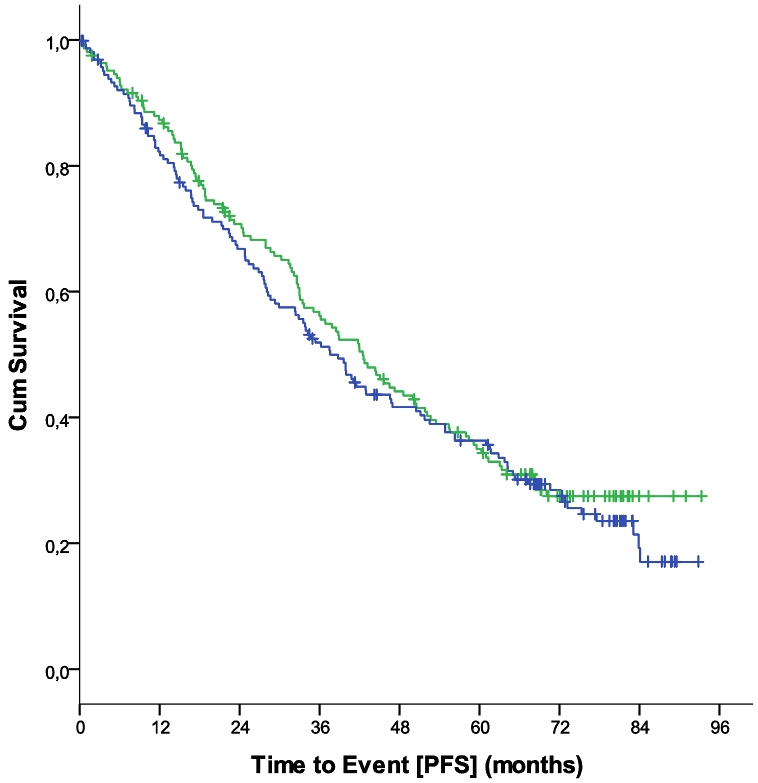

p = 0.465 (log-rank)

COX regression PFS	Univariate comparison	Hazard ratio [HR]	95% Confidence Interval		*p* value
			Lower	Upper	
**LMNA**					
High	*vs.* low	1.100	0.852	1.421	0.465

PFS	Pts, N	Events, N	Median months	2-year Survival, %	3-year Survival, %	5-year Survival, %	7-year Survival, %
**LMNB2**	**337**	**235**	**39.9**				
**≤ 6.89**	84	64	41.6	74.3	55.1	38.3	30.7
**> 6.89 & ≤ 7.16**	85	60	44.9	67.5	57.5	32.2	24.8
**> 7.16 & ≤ 7.51**	84	65	39.5	67.2	51.3	35.4	17.5
**> 7.51**	84	56	38.6	66.7	52.8	37.8	17.7

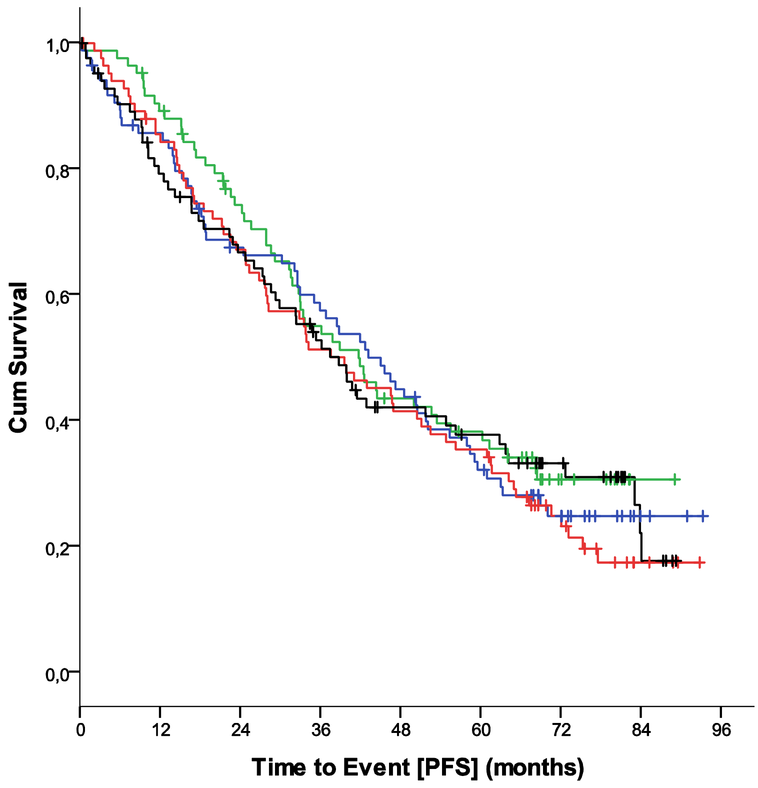

p = 0.757 (log-rank)

COX regression PFS	Univariate comparison	Hazard ratio [HR]	95% Confidence Interval		*p* value
			Lower	Upper	
**LMNB2**					
> 6.89 & ≤ 7.16	***vs.*** ≤ 6.89	1.123	0.777	1.623	0.536
> 7.16 & ≤ 7.51	***vs.*** ≤ 6.89	1.221	0.851	1.752	0.278
> 7.51	***vs.*** ≤ 6.89	1.111	0.764	1.616	0.582

Overall survival (OS) by LMNB2

COX regression OS	Univariate comparison	Hazard ratio [HR]	95% Confidence Interval		*p* value
			Lower	Upper	
**LMNA (continuous variable)**		1.201	0.898	1.607	0.218
**Study treatment**					
FCR	***vs.*** FC	0.600	0.418	0.860	0.005

OS	Pts, N	Events, N	Median months	2-year Survival, %	3-year Survival, %	5-year Survival, %	7-year Survival, %
**LMNA**	**337**	**121**	**86.3**				
≤ 6.51 (low)	169	74	86.3	89.7	85.2	74.7	55.1
> 6.51 (high)	168	47	86.1	86.5	85.9	73.3	51.1

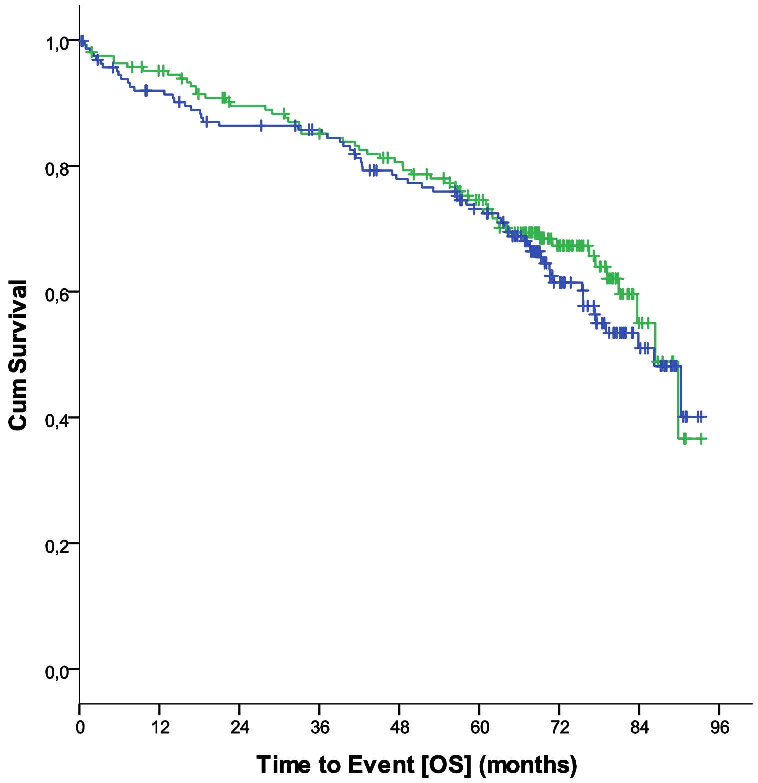

p = 0.470 (log-rank)

COX regression OS	Univariate comparison	Hazard ratio [HR]	95% Confidence Interval		*p* value
			Lower	Upper	
**LMNA**					
High	***vs.*** low	1.141	0.798	1.632	0.470

OS	Pts, N	Events, N	Median months	2-year Survival, %	3-year Survival, %	5-year Survival, %	7-year Survival, %
**LMNB2**	**337**	**121**	**86.3**				
**≤ 6.89**	84	21	86.3	95.1	92.5	84.4	58.9
**> 6.89 & ≤ 7.16**	85	35	80.8	84.4	78.2	64.4	49.5
**> 7.16 & ≤ 7.51**	84	31	NR	87.9	86.7	72.9	56.5
**> 7.51**	84	34	75.5	85.2	85.2	73.7	44.1

NR, not reached

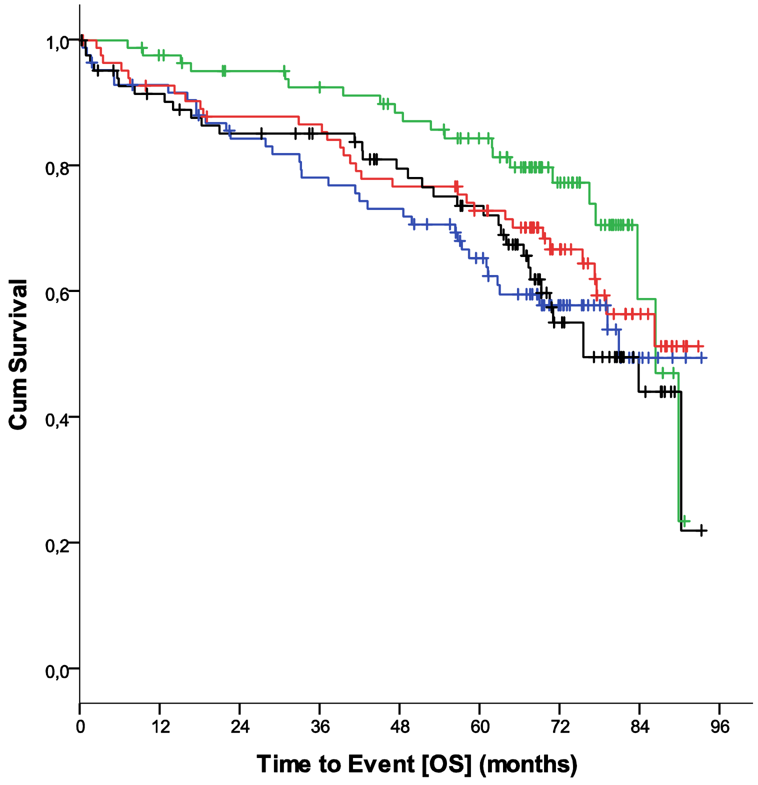

p = 0.092 (log-rank)

COX regression OS	Univariate comparison	Hazard ratio [HR]	95% Confidence Interval		*p* value
			Lower	Upper	
**LMNB2**					
> 6.89 & ≤ 7.16	***vs.*** ≤ 6.89	1.852	1.078	3.183	0.026
> 7.16 & ≤ 7.51	***vs.*** ≤ 6.89	1.414	0.812	2.463	0.221
> 7.51	***vs.*** ≤ 6.89	1.821	1.057	3.139	0.031
